# Edge dislocations, alloy composition, and grain boundaries effects on the mechanical properties in *NiCo* binary alloy

**DOI:** 10.1038/s41598-024-65437-y

**Published:** 2024-11-13

**Authors:** Md. Nadim Mahamud Nobin, Md. Lokman Ali, Md. Khairul Alam

**Affiliations:** Department of Physics, Pabna University of Science and Technology, Pabna, 6600 Bangladesh

**Keywords:** Binary alloys, Edge dislocation, Chemical composition, Grain boundary (GB), Mechanical stability, Mechanical properties, Materials science, Physics

## Abstract

In this research, we investigated the mechanical properties of NiCo binary alloy both with and without grain boundaries, across various alloy compositions. We investigated the effects of edge dislocation, alloy compositions, and grain boundaries on the mechanical properties of FCC $NiCo$ using molecular dynamics (MD) simulations. By analyzing the influence of the grain boundaries with different alloy compositions on several mechanical properties, we were able to gain a deeper understanding of how these characteristics were modified. The elastic moduli increased with increasing grain boundaries for a specific chemical composition, indicating that $NiCo$ becomes more rigid. The anisotropy factor analysis showed that $NiCo$ have a natural tendency toward anisotropy, which is further influenced by the presence of grain boundaries. Grain boundaries have a significant effect on strength and ductility across a wide range of alloy compositions. These results indicate that a deeper comprehension of these implications can aid in designing improved binary alloy with superior mechanical characteristics.

## Introduction

Ni-based superalloys have been employed in turbine engines for decades due to their superior high temperature tensile, creep, and fatigue properties^1–3^. A large number of research has been done to improve the materials performance by adding a variety of alloying elements, including Co, Cs, W, Nb, Ti, Mo, Hf etc., to the Ni-base alloys, in response to the ever-increasing requirement for materials properties^1–3^. By influencing a variety of microstructural factors, such as solid solution hardening (dislocation-solutes interaction^4,5^, stacking fault energy^5–7^, elastic moduli^8^ and lattice parameters^5,9^, strengthening interface^10^, formation of local brittle zones^11–13^, diffusion mobility^14^ and grain boundaries^15^, and tendency of segregation to grain boundaries^16,17^, those elements are known to have a significant impact on mechanical properties.

Several strategies have been implemented to enhance the mechanical properties of NiCo alloy at ambient temperature. These strategies encompass the creation of heterogeneous microstructures, the incorporation of hierarchical microstructures^18,19^, the utilization of precipitation hardening techniques^20,21^, and the adjustment of phase combinations^22,23^. Grain refining is a well-established and very effective method among several strengthening procedures^24^. Due to a significant proportion of grain boundaries (GBs) acting as obstacles to impede the movement of dislocations, nanocrystalline (NC) metals are capable of achieving an exceptionally high yield strength that exceeds that of their coarse-grained (CG) counterparts by more than tenfold^25^. Numerous methods have been developed for the preparation of binary alloys, and these alloys have shown promise in terms of their mechanical properties^26–28^. The utilization of the instrumental nano-indentation approach has been extensively employed for the assessment of the mechanical characteristics, including elastic properties^29^, hardness^30^, creep behavior^31^, and fracture toughness^32^. Experiments show that there are multiple discrete displacement bursts, or pop-ins, on the load–displacement curves at small indentation depths of several tens of nanometers or less^33,34^. The initial occurrence of pop-in events, referred to as incipient plasticity, is thought to be associated with the initiation of plastic deformation, which is governed by the production of dislocations at a stress level close to the theoretical strength of the material^35,36^. The grain boundaries (GBs) exhibit chemical complexity with localized variations in composition, including GB complexions, which can have a substantial impact on the interactions between defects and GBs^37,38^. High-volume fraction GBs have a significant effect on the nanoscale plasticity of various alloys^39,40^. Grain boundaries (GBs) have the capability to facilitate plastic deformation through several mechanisms such as GB sliding, GB migration, and grain rotation^41^. Additionally, GBs can also play an indirect role by serving as origins and destinations for dislocations^42^. Compared to GB networks in pure metals and traditional dilute alloys, it has been observed that binary alloys exhibit some unique properties^43^. A reasonable follow-up question is whether or whether the presence of GBs, in addition to the effect of chemical composition, can have any further bearing on the mechanical properties of materials. The answer to this question is not only necessary for a comprehensive understanding of mechanical properties in alloys, but also for the application of these alloys, as they are promising candidates for use in micro electro-mechanical systems (MEMS) and precision instruments, where extremely high structural robustness and health are rigorously required^44,45^. Recent theoretical and experimental work has established a linear relationship between the critical resolved shear stress (CRSS) and several atomic distortion parameters, including the mean-square atomic displacement (MSAD)^46^, the atomic volume difference^47–49^, and the local atomic strain^50^.

The main aim of this study is to establish a correlation between the CRSS and MSAD in binary alloy systems. In order to accomplish this objective, we will employ molecular dynamic (MD) simulations, specifically emphasizing direct atomistic deformation testing. This research uses three-dimensional atomic simulations to examine how the variation in atomic composition at grain boundaries (GBs) affects dislocation emission from GBs in NiCo alloy. In this research, models are initially constructed by incorporating parts of varying compositions. Following this, we proceed to include edge dislocation and grain boundaries in order to better examine their impact on mechanical properties. For the first time we have investigated the effect of alloy composition and grain boundaries on the mechanical properties of binary alloys. Two potentials, namely the embedded atomic model (EAM) potential and the Lennard–Jones (LJ) potential, have been employed in our study. Furthermore, we have conducted a comparative analysis of these two potentials, examining their impact on mechanical properties in relation to chemical compositions and grain boundaries.

## Computational methods

### Interatomic potential

The motion of defects in metallic materials, such as dislocation slides and vacancy diffusion, can be studied using the Lennard–Jones (LJ) potential, despite the fact that the many-body effect is ignored. From rare gas materials to metallic liquids and grain boundaries, this potential has been used for modeling a wide variety of phases and structural flaws. We choose to incorporate the LJ potential because it allows for straightforward manipulation of the MSAD and CRSS of the alloying systems. This same element’s LJ potential can be parameterized using the following expression.

1 $$ U_{LJ} = 4\varepsilon_{ii} \left\{ {\left( {\frac{{\sigma_{ii} }}{r}} \right)^{12} - \left( {\frac{{\sigma_{ii} }}{r}} \right)^{6} } \right\} $$

where $\varepsilon_{ij}$ is the length of the energy well and $\sigma_{ii}$ is the length that specifies the limiting length of the interatomic interaction. The LJ potential parameters can be expressed in terms of i and j for each component using Eq. (1). The values $\varepsilon_{ij}$ and $\sigma_{ii}$ can be calculated using the equations $\varepsilon_{ij} = \sqrt {\varepsilon_{ii} \varepsilon_{jj} }$ and $\sigma_{ij} = \sqrt {\sigma_{ii} \sigma_{jj} }$, respectively. In our previous study, we employed the Lennard–Jones potential characteristics of ternary equiatomic and near-equiatomic $NiCoCrCuFe$ high-entropy alloys, and their LJ potential properties were analyzed^51^. A value of 0.2 eV for $\varepsilon_{ij}$ has been chosen for MD simulations.

### Molecular dynamics (MD) simulations

Simulations of molecular dynamics (MD) run with the LAMMPS (Large-scale Atomic/Molecular Massively Parallel Simulator)^52^. The atomic interactions in a FCC $NiCo$ binary alloy are modelled with the use of the embedded atom method (EAM) interaction potential established by Li et al.^53^, which has been applied in a few recent MD studies, such as those involving dislocation propagation and motion^54,55^. On the fcc lattice, the elements are randomly arranged. The x and y directions were subjected to periodic boundary restrictions, whereas the z direction was left unrestricted for CRSS calculation. Both the lower and upper boundaries of the atomic layers were seen as rigid bodies, and a strain rate was applied to the higher rigid body at a relative velocity to the lower rigid body.

### Grain boundary model construction

**Figure 1:**
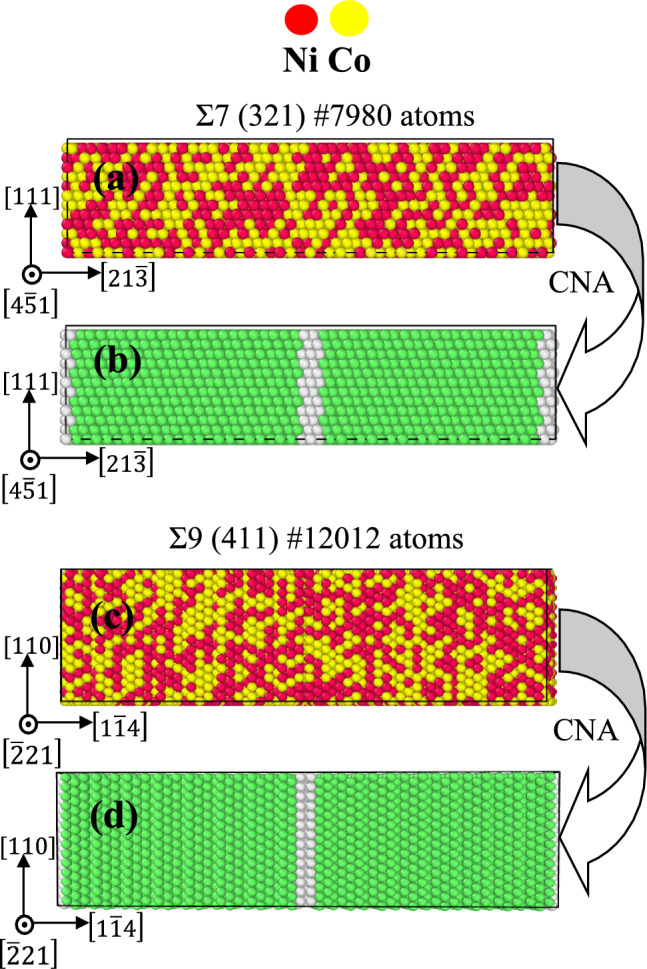
Simulation models of (**a**) Σ7 (312), (**d**) Σ9 (411) for equiatomic FCC $NiCo$ alloys, and (**b**) and (**d**), respectively, represent FCC structures for GBs Σ7 and Σ9 according to common neighbour analysis.

This study involves the construction of nanocrystalline models with various grain boundary and edge dislocation structures utilizing the molecular dynamics-based modelling software ATOMSK^56^. These simulations utilized a bicrystal model with Σ7 (312) and Σ9 (411) symmetric tilt grain boundaries, as illustrated in Fig. 1. The supercell size of the Σ7 (312) and Σ9 (411) are 10.45 nm × 3.39 nm × 2.42 nm and 11.84 nm × 3.14 nm × 3.45 nm, respectively. There are 7920 atoms in the GB Σ7 (312) model, while there are 7343 such atoms in the GB Σ9 (411) model. For $$Ni_{0.25} Co_{0.75}$$, $$Ni_{0.50} Co_{0.50}$$, and $$Ni_{0.75} Co_{0.25} $$ alloys the coordinates [21$$\overline{3}$$] [4$$\overline{5}$$1] [111], and [1$$\overline{1}$$4] [$$\overline{2}$$21] [110] of GBs Σ7 and Σ9 are aligned with the x, y, and z axes, respectively.

The original GB model predicted that $Ni$ and $Co$ atoms would be randomly distributed across lattice sites, leading to a solid solution structure with a constant atomic composition but a disordered arrangement of atoms. We used the open visualization tool (OVITO)^57^ to create a visual representation of the system. Common neighbour analysis (CNA)^58^ was used by OVITO for identifying defects, with a mean-square deviation (MSD) value of 0.1–0.15. Additionally, we used OVITO’s atomic strain tool to calculate the atomic shear strain by analyzing the strain tensor and atomic deformation gradients at each particle.

### MSAD model construction and computational method

**Figure 2:**
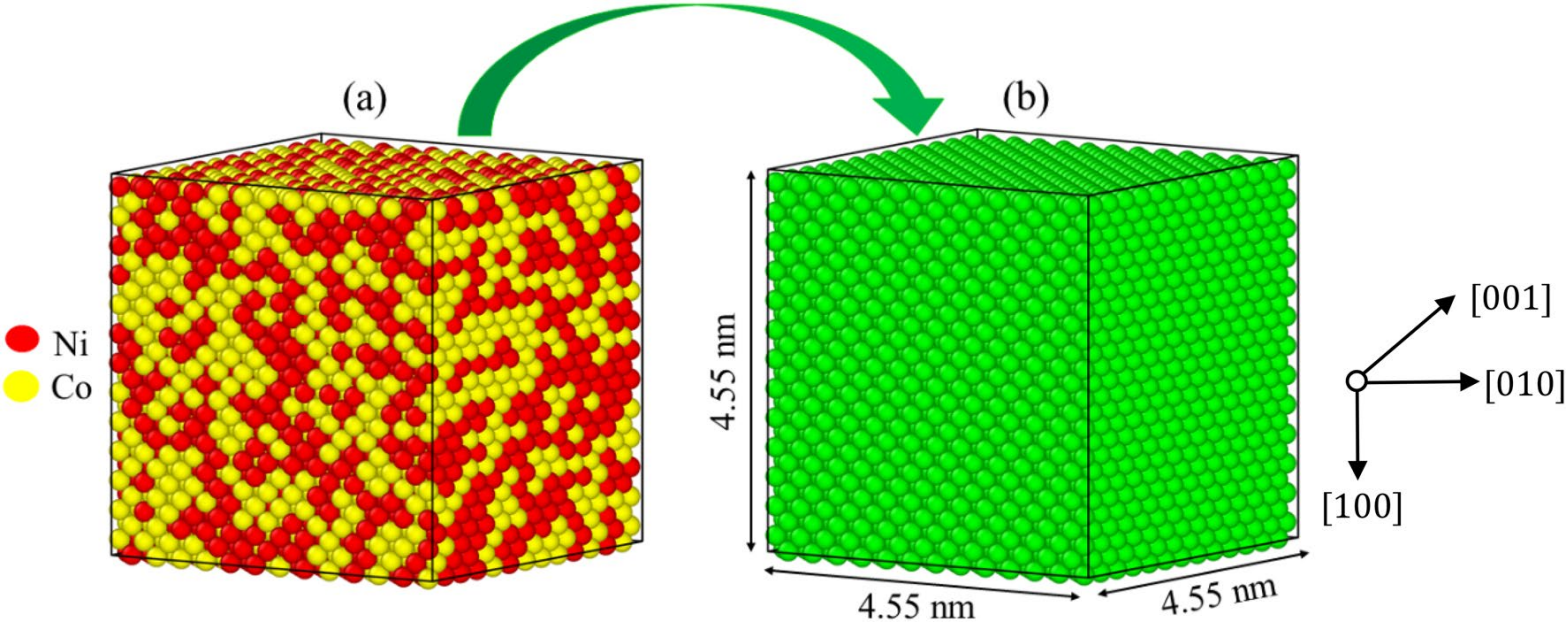
A simplified diagram of a MD simulation (**a**) the atomic distribution of a $NiCo{ }$. binary alloy with equal amounts of each element depicted by different colours (**b**) atoms are colored by the common neighbor analysis system and green atoms shows the face-centered cubic structure.

The MD simulations were performed to determine the MSAD values for different chemical composition in a three-dimensional FCC crystal structure. The three-dimensional FCC crystal comprised of 8788 atoms with a 4.55 × 4.55 × 4.55 nm^3^ supercell under periodic boundary conditions in all three directions, such as [100], [010], and [001] which was shown in Fig. 2. The exact amount of atoms for each element was randomly distributed over the FCC lattice. The following method was used to calculate the MSAD of the LJ alloy systems. To begin, (a) the model was relaxed using the volumetric supercell ensemble of NTP to maintain a fixed number of atoms (N), temperature (T), and pressure (P). In order to generate a deformed atomic structure that did not follow the FCC lattice, (b) atomic structure relaxation without supercell shape relaxation was carried out. After allowing the atomic structure to relax (b), the resulting atomic displacement was used in the following equation to calculate MSAD values^59^:

$$ {\text{MSAD}} = \sum x_{i} (R_{i} - \overline{R}^{2} ) $$

*R* is the average nearest-neighbour distance between each pair of elements, where $$R_{i} $$ and $$x_{i}$$ are the nearest-neighbor distance and the corresponding proportion, respectively. To represent the wide range of alloys with varying MSADs, we selected the appropriate potential parameters. In Table S1, we see the possible parameters for the $$Ni_{0.25} Co_{0.75}$$, $$Ni_{0.50} Co_{0.50}$$, and $$Ni_{0.75} Co_{0.25}$$ alloy systems with varying MSADs. The values for $$\varepsilon_{ij}$$ were adjusted to 0.2 eV. To determine the MSAD for each alloy, ten alternative atomic structures were simulated with the same energy and alloy composition.

### CRSS model construction and computational method

**Figure 3:**
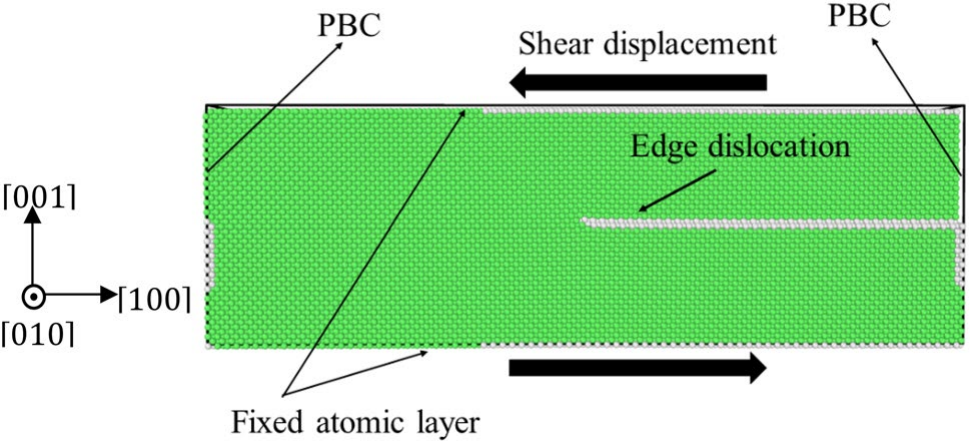
A schematic illustration depicting the required boundary conditions and a single-edge dislocation within the simulated supercell.

Figure 3 shows the simulated supercell used for CRSS computation. It is made up of FCC lattice structures along x = [100], y = [010], and z = [001], measuring 27.99 × 10.49 × 8.75 nm^3^ and containing around ~ 240,000 atoms. A single edge dislocation was created manually on the [001] plane. In the x and y directions, periodic boundary restrictions were imposed, whereas the z direction was left unconstrained. The shear strain was provided by treating the three atomic layers of the upper and lower borders as rigid bodies and then applying incremental relative displacement of 0.005 nm from the upper rigid body to the lower rigid body. After each little relative motion, the atomic structure was relaxed using the conjugate gradient approach, with a force tolerance of 0.001 eV/Å. The response force acting on the rigidly shifting body’s cross-sectional area along the [001] direction is used to get an approximation of the applied shear stress. It is possible to estimate the CRSS as the shear stress operating at the point where the dislocation begins to glide.

## Results and discussion

### Lattice distortion, yield strength, MSAD and CRSS

**Figure 4:**
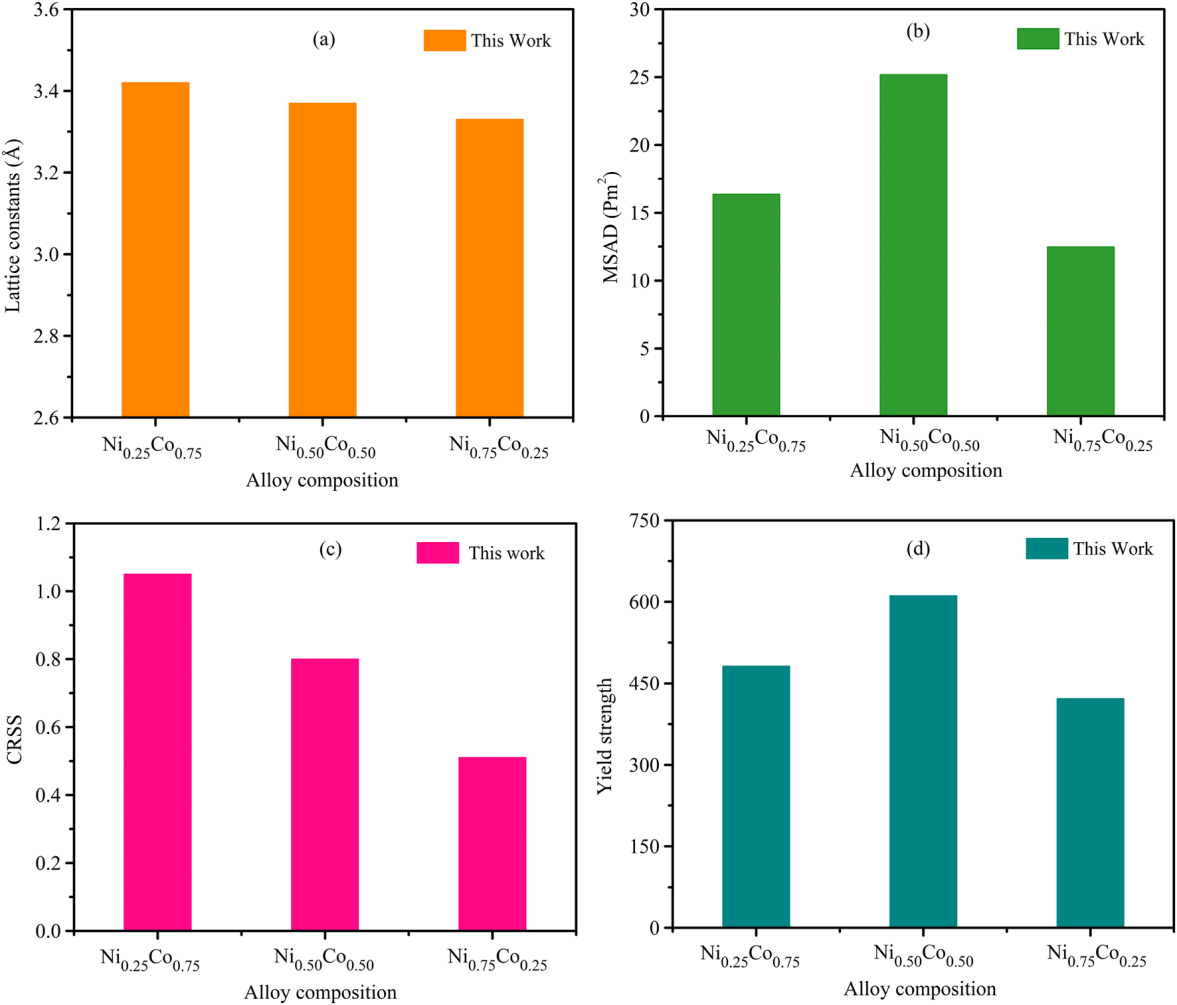
Influence of alloy compositions on the MD simulations of (**a**) lattice constants, (**b**) MSAD values, (**c**) CRSS and (**d**) yield strength of several chemical compositions of $NiCo$ binary alloys.

Our investigated compound $NiCo$ demonstrates the face-centred cubic phase. Lattice parameters calculated for various alloy compositions are listed in Table S1 and depicted in Fig. 4a, which clearly shows a decrease in lattice constant values as Ni concentration rises in the alloy composition. A stronger atomic link may be inferred from this observation. Increased atomic connections and reduced interatomic spacing may produce changes to the alloy’s mechanical characteristics.

Lattice distortion is an essential material features. Lattice distortion is often proportional to the atomic size of the constituent atoms. The MSAD is a standard method used to examine $NiCoCr$ and other reported ternary systems for atomic distortion^60^. Lattice distortion is greatly reduced in pure Ni and Al compared to $CrCoNi$ alloy^61^. Tong et al.^62^ used X-ray diffraction to compare $CrCoNi$, $CrCoFeNi$, and $NiCoCrFeMn$ HEA, and found that $CrCoNi$’s lattice distortion is similar but more pronounced than, that of $NiCoCrFeMn$ HEA. MSAD was used in this study to analyze the degree of lattice distortion in a range of alloy compositions. Since the distorted lattice alone determines the resistance to dislocation movement, and since the tendency to yield strength may be predicted by analyzing the appropriate MSAD data, so this assessment is crucial. Table S1 shows the MSAD, CRSS, and yield strength of $NiCo$ binary alloys as calculated by MD simulation.

The MSAD value provides a helpful scaling factor for estimating the yield strength. According to the research of Okamoto et al.^59^, the square root of the relevant MSAD values is proportional to the yield strength at 0 K normalized by shear modulus of FCC alloys. MSAD value can be calculated using the following equation^59^:

$$ \frac{{\sigma_{YS} }}{\mu } = k \cdot \sqrt {MSAD} $$

where, k is a constant. The value of k was determined by Sohn et al. to be 1.3817^63^. This is significant because the distorted crystal alone determines the resistance to dislocation motion, by accurately identifying the suitable values of the MSAD, it becomes possible to anticipate the trends in yield strength^59^.

In this case, the MSAD values are enhanced with $$Ni_{0.50} Co_{0.50} $$ alloy composition. However, the worth of MSAD is diminished by $$Ni_{0.25} Co_{0.75}$$ and $$Ni_{0.75} Co_{0.25 }$$ composition. In general, we find that the MSAD values in the $$NiCo$$ alloy are more influenced by the equiatomic alloy composition than by any other alloy composition. Therefore, it has been established that the introduction of $$Ni_{0.25} Co_{0.75}$$ and $$Ni_{0.75} Co_{0.25 }$$ composites results in less atomic distortion, the primary cause of a reduction in yield strength. The MSAD values and yield strength of multiple models of NiCo with varying chemical composition are shown from MD simulations in Fig. 4b,d. Chemical compositions were shown to have a larger impact on yield strength, and the MSAD parameter was found to be an essential scaling quantity for estimating yield strength.

Figure 4c demonstrates that the estimated CRSS values value falls when the alloy composition is varied. We found the highest and lowest CRSS value for $Ni_{0.25} Co_{0.75}$ and $Ni_{0.75} Co_{0.25 }$ alloy composition, respectively. We hope that this information will be helpful for the advancement of high-throughput, high-strength of NiCo alloy design.

### Elastic properties

**Figure 5:**
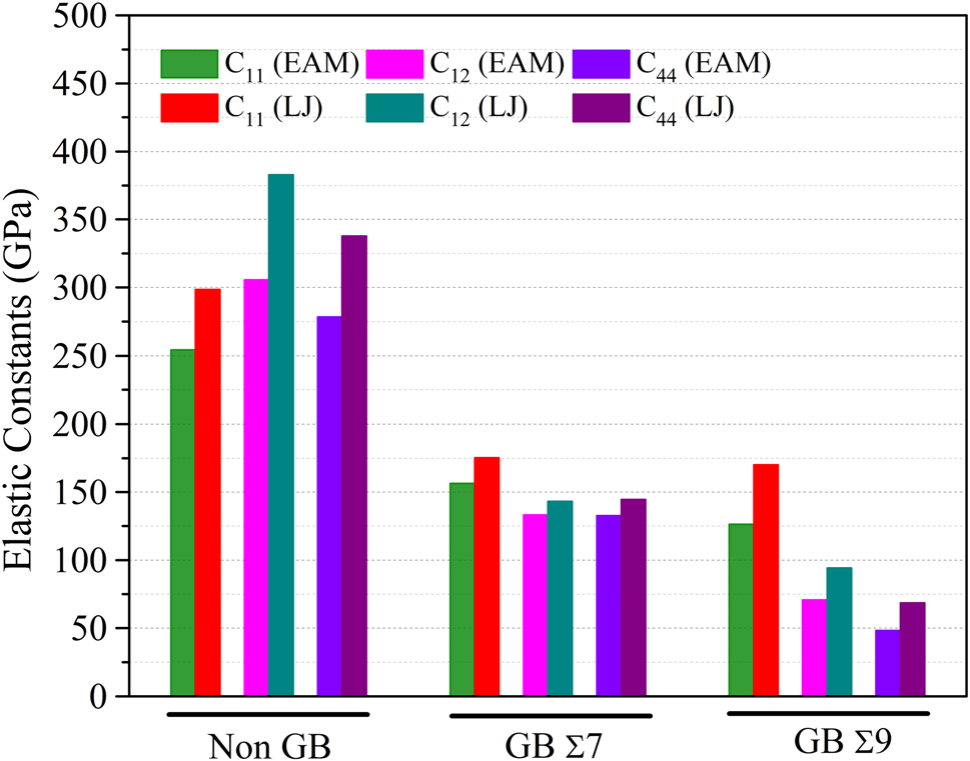
The effects of grain boundary on elastic constants $C_{ij}$ (GPa) of $NiCo$ binary alloys at Non-GB, GB Σ7 and GB Σ9 for $Ni_{0.25} Co_{0.75}$ alloy composition.

**Figure 6:**
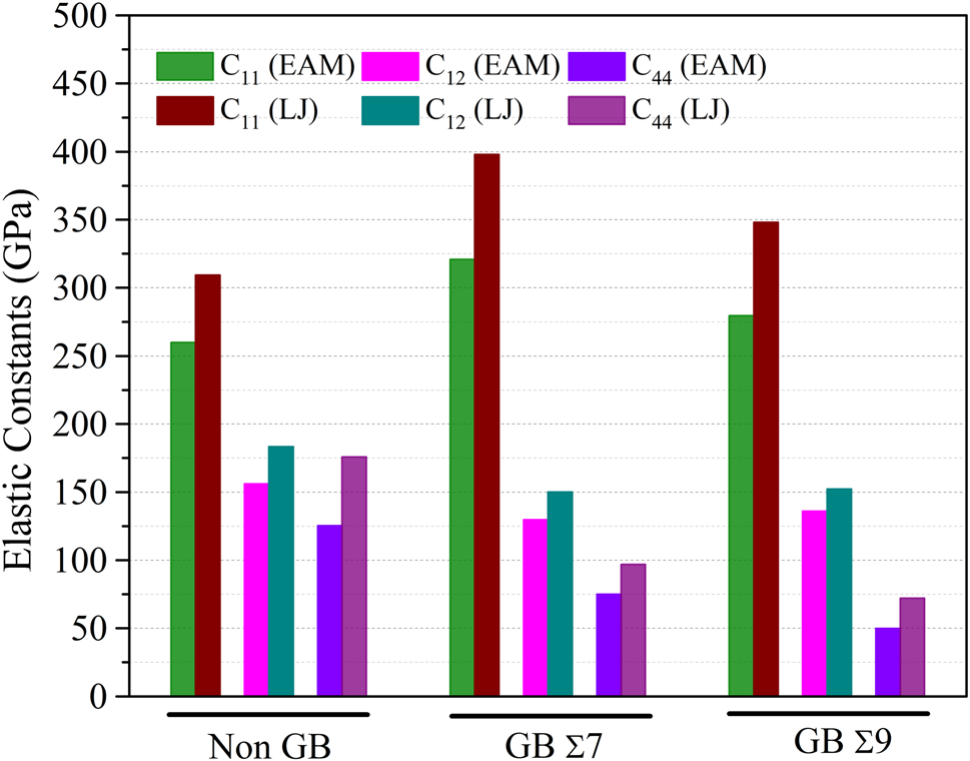
The effects of grain boundary on elastic constants $C_{ij}$ (GPa) of $NiCo$ binary alloys at Non-GB, GB Σ7 and GB Σ9 for $Ni_{0.50} Co_{0.50}$ alloy composition.

**Figure 7:**
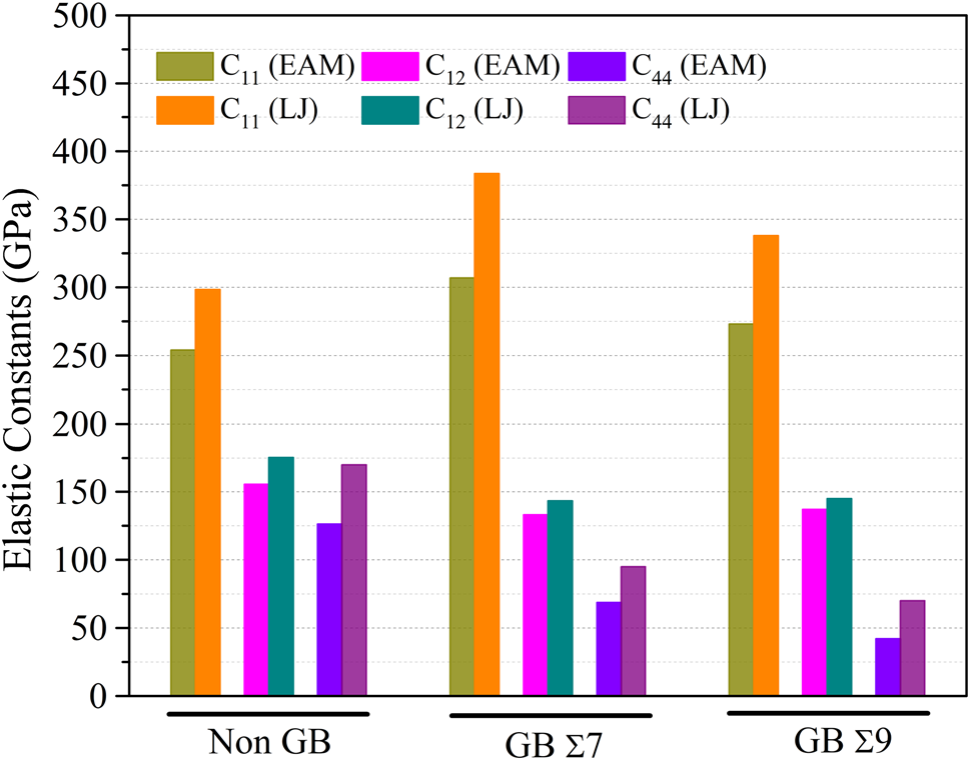
The effects of grain boundary on elastic constants $C_{ij}$ (GPa) of $NiCo$ alloys at Non-GB, GB Σ7 and GB Σ9 for $Ni_{0.75} Co_{0.25}$ alloy composition.

For describing the bonding patterns and mechanical characteristics of solids, the elastic constants $$C_{ij}$$ are crucial. These constants additionally describe the physics behind the mechanical stability of a solid and the correlation between the phonon spectrum and the Debye temperature of a crystal. Mechanical characteristics and structural stability are greatly affected by a substance’s elastic constants. These constants reveal the amount of strain-induced deformation and subsequent recovery to the original shape of a material when the strain is removed, linking the dynamical and mechanical behavior of crystals. For cubic materials, the elastic constants $$C_{11}$$, $$ C_{12}$$ and $$C_{44}$$ are all independent numbers. The shear deformation resistance of a material is expressed by the elastic constant $$C_{44}$$, the shape elasticity by the elastic constant $$ C_{12}$$, and the length elasticity by the elastic constant $$C_{11}$$. The computed values for $$C_{11}$$, $$ C_{12}$$ and $$C_{44}$$, and the Cauchy pressure ($$ C_{12}$$–$$C_{44}$$) are displayed in Tables S2, S3, S4. For a cubic crystal to be mechanically stable, its elastic constants $$C_{44}$$ and ($$ C_{12}$$–$$C_{44}$$) must both be positive. The computed elastic constants for these conditions for different alloy composition such as $$Ni_{0.25} Co_{0.75}$$, $$Ni_{0.50} Co_{0.50}$$, and $$Ni_{0.75} Co_{0.25}$$ are presented in Tables S2, S3, S4, respectively. Different types of $$NiCo$$ binary alloys, such as those without grain boundaries and those with Σ7 and Σ9 boundaries with various alloy composition exhibit different ranges of elastic constants $$C_{ij}$$, as shown in Figs. 5, 6 and 7. In order to ascertain a lattice’s mechanical stability, Born stability criteria are applied^64^; these criteria are often expressed in terms of $$C_{ij}$$ and are hence reliant on free energy considerations. Spinodal, shear, and Born conditions for a cubic crystal are $$C_{11} + 2C_{12}$$ > 0, $$C_{11} - C_{12}$$ > 0, and $$C_{44}$$ > 0^65^. The estimated elastic constants show that the chemical under study satisfies the well-known Born stability criterion, making it mechanically stable even for Σ7 and Σ9.

Numerous grain boundary types and computational models have been used to calculate the elastic constants of $$Ni_{0.25} Co_{0.75}$$, $$Ni_{0.50} Co_{0.50}$$, and $$Ni_{0.75} Co_{0.25} $$ alloys. The information in Tables S2 to S4 emphasizes the significance of taking into account the computational model and grain boundary type when evaluating the mechanical properties of these alloys. These results provide insightful information on the anisotropic behavior and deformation processes of various alloys composition, which may have implications for their use in engineering and materials research.

**Figure 8:**
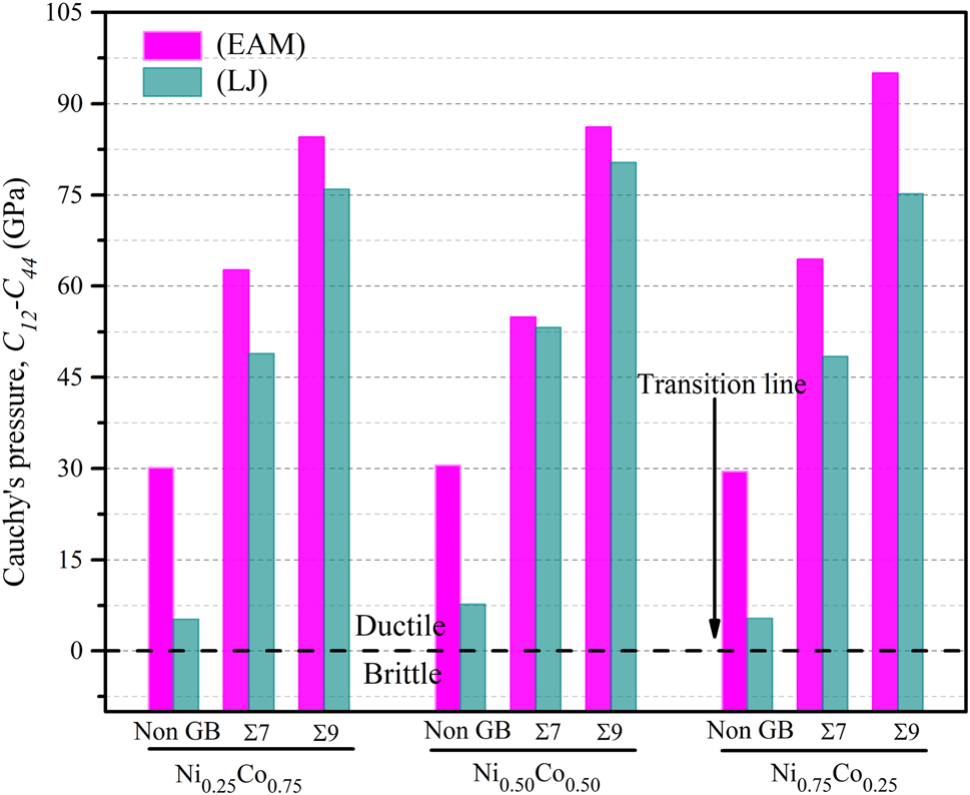
The influence of alloy composition and the grain boundary on Cauchy’s pressure in NiCo binary alloys.

Additionally, the Cauchy pressure ($C_{12}{-}C_{44}$) can be used to determine the degree of brittleness and ductility. If ($C_{12}{-}C_{44}$) is negative (positive), the material should be brittle (ductile)^66^. The outcomes of our calculations for Cauchy’s pressure are presented in Tables S2–S4. Figure 8 depicts a bar diagram for different models with $Ni_{0.25} Co_{0.75}$, $Ni_{0.50} Co_{0.50}$, and $Ni_{0.75} Co_{0.25}$ alloy compositions, demonstrating the influence of chemical composition and grain boundaries on Cauchy’s pressure in $NiCo$ alloys. The enhanced ductility of $NiCo$ binary alloys is evident in the observed changes in the model’s ($C_{12}{-}C_{44}$) values, which progressively increase when the amount of Ni in the alloy composition is augmented, particularly in the cases of Non GB, GB Σ 7 and GB Σ 9.

### Alloy composition and GB effects on mechanical properties

In the field of engineering, the concept of elastic moduli is of crucial significance. Mechanical characteristics of $NiCo$ alloy were calculated using the Voigt–Reuss–Hill (VRH) equating method^67^, which gave us Young’s modulus $E$, bulk modulus $B$, Poisson’s ratio $\nu$, and shear modulus. The Voigt (V) approximations provide upper and lower limits on B and G, while the Reuss (R) approximations quantify these boundaries. Hill suggests that using the arithmetic mean of the Reuss and Voigt limits is a reliable way to get the effective shear and bulk moduli. Voigt–Reuss–Hill (VRH) averaging techniques are used to calculate the elastic moduli of NiCo binary alloys^52^.

Table S2 shows the calculated values for the different elastic properties of $NiCo$ alloys. The $G$ value quantifies the material’s resistance to plastic deformation, while the $B$ value quantifies its resistance to fracture. When a substance is stretched or compressed in one direction, its $E$ value indicates how much resistance it will have to the change in length. In comparison to other metals, the bulk modulus ($B$) and Young’s modulus ($E$) of a variety of $NiCo$ binary alloys are quite small. When the $G$ value is less than the $B$ value, plastic distortion is the main thing that determines the mechanical strength. The value of $B$ is directly proportional to the volume of the cell^68^. By determining the binding energy or cohesion energy of the atoms in the crystal lattice, we can estimate the average atomic bond strength in a material using the parameter $B$. On the other hand, if $G$ is large, then there are many strong directed bonding contacts between the atoms^69,70^.

**Figure 9:**
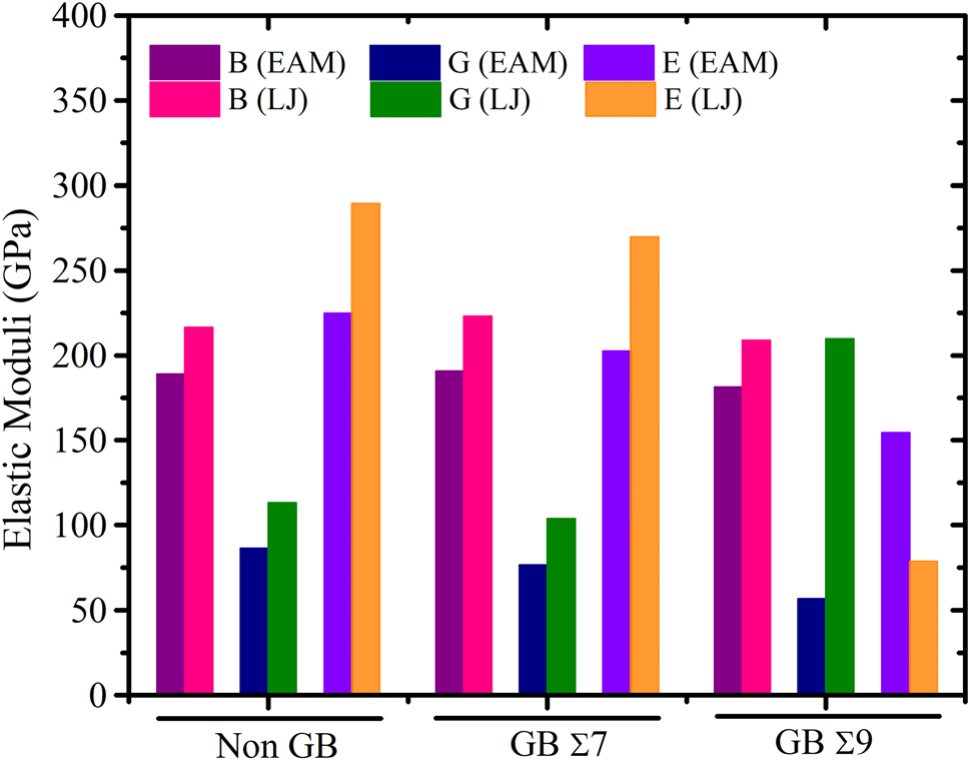
Grain boundary impact on the shear modulus, the bulk modulus, and the Young’s modulus of $NiCo$ binary alloys for Non-GB, GB Σ7, and GB Σ9 of $Ni_{0.25} Co_{0.75}$ alloy composition for two different potentials.

**Figure 10:**
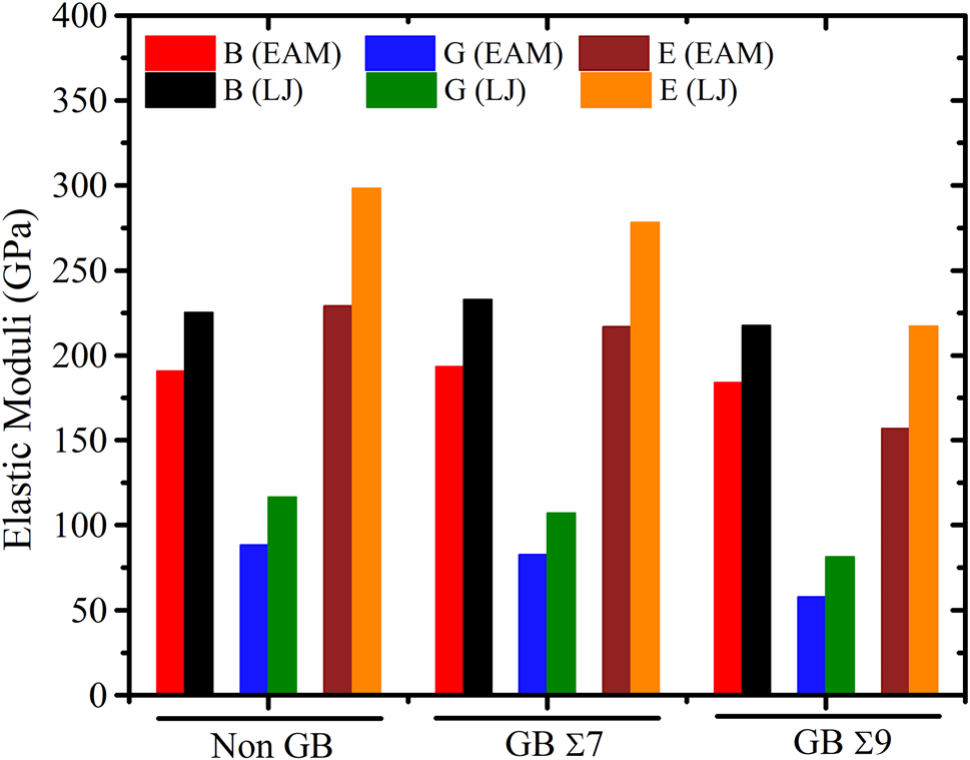
The influence of grain boundary on the shear modulus, the bulk modulus, and the Young’s modulus of $NiCo$ binary alloys of $Ni_{0.50} Co_{0.50}$ alloy composition for two different potentials.

**Figure 11:**
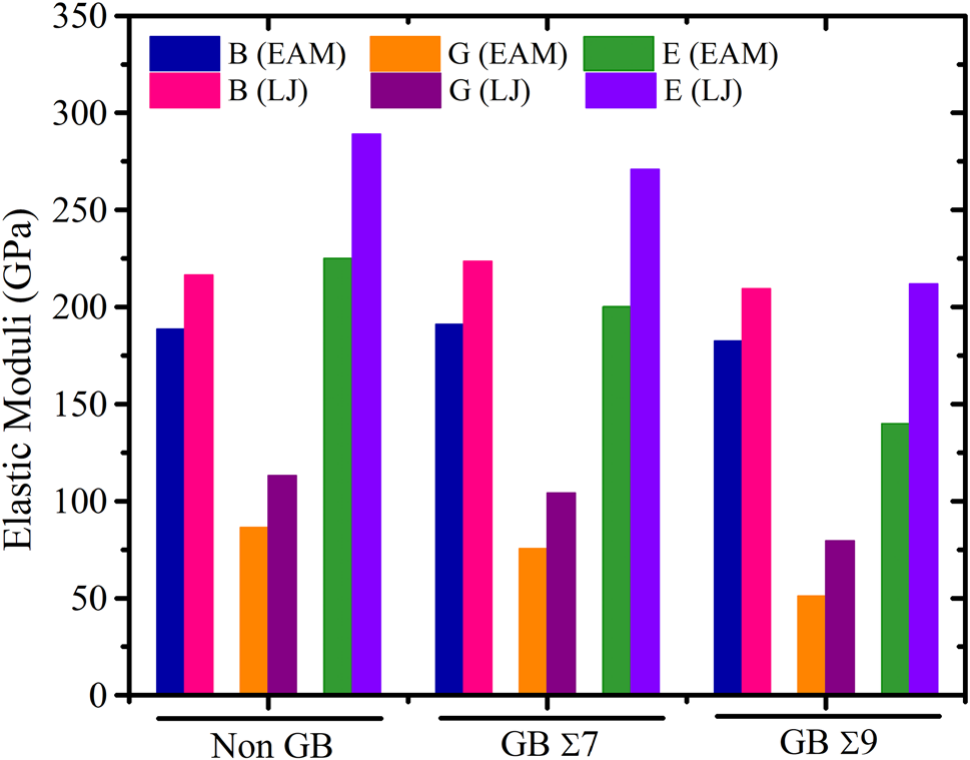
The influence of grain boundary on the shear modulus, the bulk modulus, and the Young’s modulus of $NiCo$ binary alloys of $Ni_{0.75} Co_{0.25}$ alloy composition for two different potentials.

Elastic moduli for $NiCo$ alloys are listed in Table 1. The effects of grain boundaries on the bulk modulus *B* (in GPa), shear modulus *G* (in GPa), and Young’s modulus $E$ of $NiCo$ alloys are graphically represented in Figs. 9, 10 and 11 for $Ni_{0.25} Co_{0.75}$, $Ni_{0.50} Co_{0.50}$, and $Ni_{0.75} Co_{0.25}$ alloy compositions, respectively. The graph depicts three distinct conditions: no grain boundary, GB Σ 7, and GB Σ 9.

**Table 1:** Calculated elastic moduli of $NiCo$ binary alloys.

Alloy composition	Grain boundary type	Potential	*B*	*G*	*E*	*B/G*	*A*	*ν*
$Ni_{0.25} Co_{0.75}$	Non GB	EAM	189.03	86.36	224.84	2.19	2.58	0.30
		LJ	216.48	113.25	289.37	1.91	2.76	0.28
	Σ7	EAM	190.72	76.50	202.44	2.49	0.82	0.32
		LJ	223.12	103.84	269.68	2.15	0.79	0.29
	Σ9	EAM	181.25	56.88	154.49	3.19	0.66	0.36
		LJ	208.88	78.66	209.66	2.66	0.71	0.33
$Ni_{0.50} Co_{0.50}$	Non GB	EAM	190.64	88.10	229.03	2.16	2.41	0.29
		LJ	225.32	116.49	298.10	1.93	2.79	0.28
	Σ7	EAM	193.42	82.54	216.78	2.34	0.78	0.31
		LJ	232.80	106.99	278.34	2.18	0.78	0.30
	Σ9	EAM	183.88	57.73	156.79	3.19	0.70	0.36
		LJ	217.52	81.39	217.09	2.67	0.73	0.33
$Ni_{0.75} Co_{0.25}$	Non GB	EAM	188.46	86.42	224.89	2.18	2.57	0.30
		LJ	216.36	113.14	289.04	1.91	2.76	0.28
	Σ7	EAM	190.98	75.46	200.03	2.53	0.79	0.33
		LJ	223.39	104.29	270.75	2.14	0.79	0.29
	Σ9	EAM	182.31	50.98	139.89	3.58	0.62	0.37
		LJ	209.34	79.52	211.74	2.63	0.72	0.33

The elastic moduli (bulk modulus, shear modulus, and Young’s modulus) of $NiCo$ binary alloys are substantially affected by alloy composition and grain boundary type, with variations observed under different potential types, EAM and LJ. The composition of an alloy, especially when it is well-balanced as in $Ni_{0.50} Co_{0.50}$, can have a significant effect on its stiffness and resistance to deformation, leading to greater values of elastic moduli as compared to. Conversely, this values are often lower with the presence of grain boundaries because of the introduction of defects and disruptions in the crystalline structure, with GB Σ 9 barriers having the most noticeable influence. It is important to note that the choice of potential type also matters, with LJ potential typically resulting in larger moduli values than EAM. These results highlight the significance of adapting the mechanical properties of these alloys for specific applications by considering both alloy composition and grain boundary type within the framework of potential selection, hence directing materials design and engineering applications.

One of the most significant aspects of engineering is ductility, which is a fundamental parameter of mechanical properties that may be studied in more depth using elastic parameters. As a measure of a material’s elasticity, Poisson’s ratio $\nu$ can be used to gain insight into its ductility and shear stability. In addition, it is used to define the binding strengths that exist between particles in a solid^71^. The parameter $\nu$ must be between 0.25 and 0.50 for a material to be considered a central force solid. The value of is more than 0.26 in ductile materials and less than 0.26 in brittle ones^72^. The ductile/brittle transition can be anticipated with the use of Pugh’s ratio B/G. If the critical value is greater than 1.75, the material is ductile, but if it is less than 1.75, it is brittle^73^. As an indicator of a material’s ductility or brittleness, the Cauchy pressure can be determined for FCC systems with the formula ($C_{12}{-}C_{44}$). As the Cauchy pressure is increased (decreased), the material’s hardness and bonding characteristics will change accordingly.

**Figure 12:**
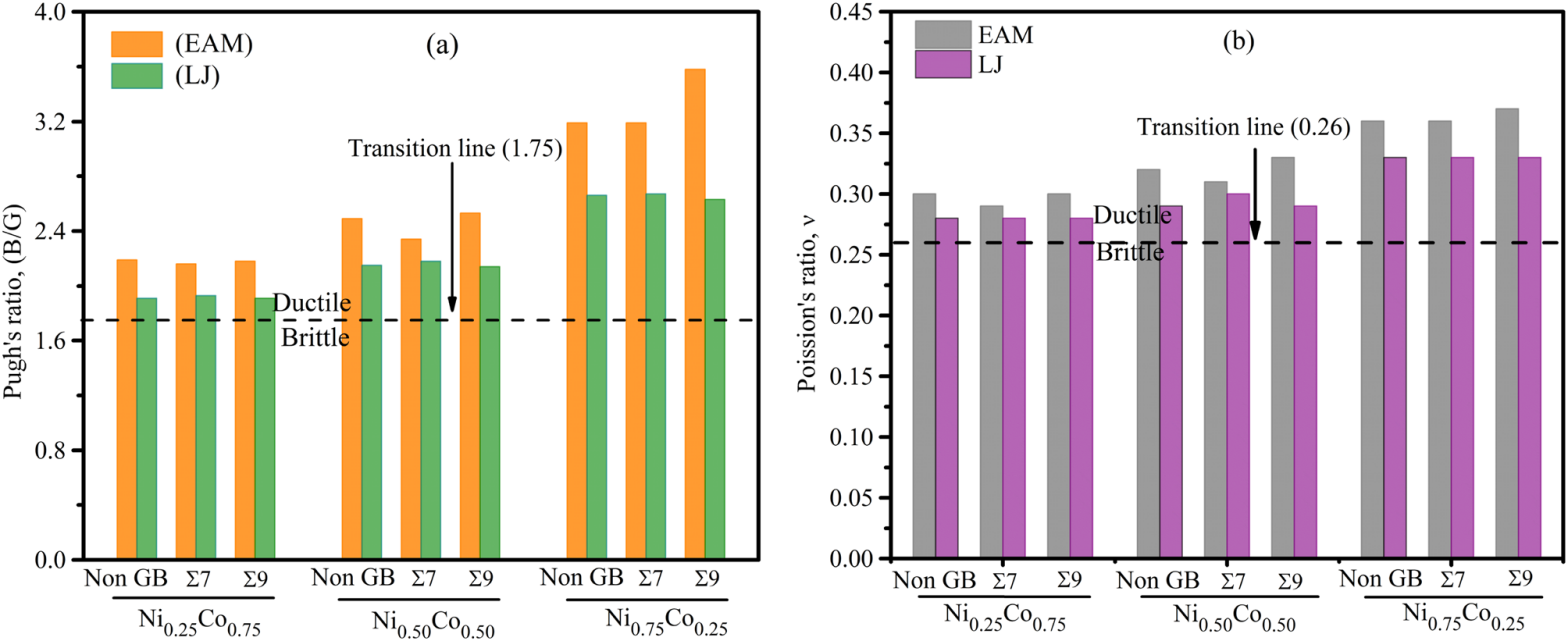
The influence of the alloy composition and grain boundary on (**a**) Pugh’s ratio and $\left( {B/G} \right)$, (**b**) Poisson’s ratio ($\nu$) in $NiCo$ alloys.

The influence of the alloys composition and grain boundary on ductility in $NiCo$ binary alloy are shown in Fig. 12a,b. The Poisson’s and Pugh’s ratios for GB Σ7 demonstrate comparatively lower values as compared to non-grain boundaries for all alloy compositions. Nevertheless, the ratios exhibit an upward trend when taking into account the GB Σ9. The influence of GB Σ9 is more significant compared to GB Σ7. Moreover, it is often observed that alloy compositions of nickel and cobalt ($Ni_{0.25} Co_{0.75}$) typically demonstrate lower values of B/G and ν. On the other hand, compositions that are more extreme, such as $Ni_{0.75} Co_{0.25}$, tend to exhibit higher values of B/G and ν. The results obtained from our research demonstrate that grain boundaries and alloy composition have a substantial role in influencing the ductility of FCC $NiCo$ binary alloys. Consequently, this offers the potential to customize and optimize the desired mechanical characteristics. Hence, it is imperative to examine the influence of grain boundaries on the ductility of materials.

The Kleinman parameter, a dimensionless quantity, typically ranges from 0 to 1 numerically. The finding indicates the comparative strength of bonding in bending and stretching. This measure assesses the capacity of a substance to withstand tensile and flexural forces^74,75^. As the extent of bond stretching decreases, the numerical value tends towards 1; conversely, as the extent of bond bending decreases, the numerical value tends towards 0. Tables S5 and S6 presents the observed influence of grain boundaries on the Kleinman parameter within $NiCo$ alloys. The results suggest that the material demonstrates a bonding mechanism defined by bending, whereas the existence of grain boundaries, specifically GB Σ7 and GB Σ9, contributes to its reduction.

The machinability index $\mu_{M}$, is crucial in manufacturing processes because it provides information about the reduction strength, optimal machine setup, and plastic strain of material. One of the most important metrics used to assess productivity in the engineering fields, symbolized as $\mu_{M}$ = *B/*$C_{44}$. High $\mu_{M}$ values are associated with dry lubrication and simple customization^76^. Diamond, usually acknowledged as the hardest substance on earth, is said to have a value of 0.8, while aluminum, widely recognized as being quite soft, is reported to have a value of 2.6^77^. Tables S5 and S6 shows the $\mu_{M}$ values for several $NiCo$ models. Within the range of alloy compositions, which include various combinations of chemical elements such as nickel (Ni) and cobalt (Co), it becomes apparent that some compositions, characterized by machinability index values of 4.34, 3.76, and 3.69, for (EAM potentials) have a greater propensity for facilitating machining processes. This observation implies that the machinability of alloys can be improved through the utilization of particular combinations of chemical components. Furthermore, it has been shown that the kind of grain boundary, such as non-grain boundary, GB Σ7, and GB Σ9, has an impact on machinability to a certain extent. Generally, GB Σ9 configurations tend to exhibit higher values of the machinability index in comparison to Non GB and GB Σ7. By selecting materials with the proper chemical composition for ease of machining while maximizing manufacturing processes, producers and engineers can benefit from this complete data.

### Chemical composition and GB effects on hardness values

In order to calculate the Vickers hardness $H_{v}$ of face-centered cubic $NiCo$ alloys, a theoretical model developed by Chen et al.^77^ can be used. Semi-empirical correlations are used to calculate the hardness of the alloys^78^. These correlations map Vickers hardness and macroscopic models for hardness prediction, including parameters such as E, G, B, and $\nu$, and G/B. $NiCo$ is a binary alloy, and the calculated hardness values are displayed in Tables 2 and 3. The hardness value for NiCo alloys reveals consistent trends for both EAM and LJ potential. Hardness generally decreases as the grain boundary type transitions from non-grain boundary to GB Σ7 to GB Σ9. Alloy compositions with a balanced ratio of Ni and Co ($Ni_{0.50} Co_{0.50}$) consistently exhibit higher hardness values compared to the extreme compositions ($Ni_{0.25} Co_{0.75}$ and $Ni_{0.75} Co_{0.25}$) across various grain boundary types. Moreover, the choice of grain boundary type, particularly GB Σ9, has a notable influence on hardness, consistently yielding the lowest hardness values in both EAM and LJ potential scenarios. These findings hold significance for materials designing and engineering applications.

**Table 2:** The calculated hardness of $NiCo$ binary alloys for EAM potential.

Alloy composition	Grain boundary type	*H*_1_	*H*_2_	*H*_3_	*H*_4_	*H*_5_	*H*_6_	*H*_7_
$Ni_{0.25} Co_{0.75}$	Non GB	18.20	13.65	12.74	14.28	12.38	9.69	11.53
	Σ7	18.37	12.29	11.28	12.85	10.63	8.67	9.20
	Σ9	17.45	9.38	8.39	9.81	7.16	6.22	5.30
$Ni_{0.50} Co_{0.50}$	Non GB	18.36	13.90	12.99	14.54	12.69	10.34	12.43
	Σ7	18.63	13.16	12.17	13.77	11.70	9.35	10.48
	Σ9	17.71	9.52	8.52	9.96	7.31	6.31	5.38
$Ni_{0.75} Co_{0.25}$	Non GB	18.15	13.65	12.75	14.28	12.39	9.66	11.53
	Σ7	18.39	12.14	11.13	12.70	10.45	8.14	8.52
	Σ9	17.56	8.49	7.52	8.88	6.12	5.77	4.42

**Table 3:** The calculated hardness of $NiCo$ binary alloys for LJ potential.

Alloy composition	Grain boundary type	*H*_1_	*H*_2_	*H*_3_	*H*_4_	*H*_5_	*H*_6_	*H*_7_
$Ni_{0.25} Co_{0.75}$	Non GB	20.85	17.56	16.70	18.37	17.13	20.85	16.58
	Σ7	21.49	16.37	15.32	17.12	15.47	21.49	14.63
	Σ9	20.12	12.73	11.60	13.31	11.02	20.12	8.93
$Ni_{0.50} Co_{0.50}$	Non GB	21.70	18.09	17.18	18.93	17.71	21.70	17.08
	Σ7	22.42	16.90	15.78	17.67	16.03	22.42	14.27
	Σ9	20.95	13.18	12.01	13.79	11.50	20.95	9.25
$Ni_{0.75} Co_{0.25}$	Non GB	20.84	17.54	16.69	18.35	17.12	20.84	16.56
	Σ7	21.51	16.43	15.38	17.19	15.55	21.51	14.69
	Σ9	20.16	12.85	11.73	13.45	11.17	20.16	9.02

### GB effects on direction dependences

In solid state physics and mechanical engineering, the anisotropy factor is one of the most important things to know about a material. A study of the anisotropy factor is important for figuring out how to make things last longer because it explains why different crystallographic orientations have different bonding properties. It also shows how flexible a system is, if it has internal cracks or micro cracks, and how stable it is mechanically. Table 4 shows the calculated anisotropy factors for the different grain boundaries with $Ni_{0.25} Co_{0.75}$, $Ni_{0.50} Co_{0.50}$, and $Ni_{0.75} Co_{0.25}$ alloys composition. $A^{G}$ and $A^{B}$ have numbers between 0 and 1, where 0 means the material is isotropic and 1 means it anisotropic. During our study, we found that the $A^{G}$ values for grain boundaries Σ7 and Σ9 are a little bit higher than 0, which suggests that crystals have anisotropy. But the number of $A^{B}$ becomes 0, which shows that the crystal is isotropic. Other anisotropic parameters with values greater than 0 like $A^{U}$ and $A^{eq}$ also add to the model’s non-uniform nature. Using the log Euclidean method^79^, one can get the global Log-Euclidean index, which is written as $A^{L}$. Table 4 shows the values of $A^{L}$ that were calculated for the different grain borders. Most people know that the parameter $A^{L}$ is a very important part of the study of anisotropy. Most of the time, the number of $A^{L} \user2{ }$ is between 0 and 10.27^79^. About 90% of solid things have $A^{L}$ values that are less than one^79^. If a material has full isotropy, the value in question must have a magnitude of zero, while any value other than zero shows that it has anisotropy. We found that the global Log-Euclidean index, $A^{L}$, was less than 1 for the Σ7 and Σ9 grain boundaries with $Ni_{0.25} Co_{0.75}$, $Ni_{0.50} Co_{0.50}$, and $Ni_{0.75} Co_{0.25}$ alloys composition. When non-grain limits are taken into account, however, the value of this parameter goes up. Both EAM and LJ potential showed the same trend. The results show that anisotropy is present in the different alloy composition of $NiCo$ alloys.

**Table 4:** The effect of alloy composition and grain boundaries on Voigt and Reuss values of $$C_{44}^{R} $$ and $$C_{44}^{V }$$(in GPa), $$A^{U}$$, $$A^{eq} ,\;A^{G}$$, $$A^{B} ,$$ and $$A^{L}$$ of $$ NiCo $$ binary alloys.

Alloy composition	Grain boundary type	Potential	$C_{44}^{R}$	$C_{44}^{V}$	$A^{U}$	$A^{eq}$	$A^{G}$	$A^{B}$	$A^{L}$
$Ni_{0.25} Co_{0.75}$	Non GB	EAM	25.79	43.76	1.16	2.58	0.10	0.00	1.18
		LJ	33.27	60.23	1.35	2.76	0.12	0.00	1.33
	Σ7	EAM	25.38	26.10	0.05	1.22	0.00	0.00	0.06
		LJ	34.38	35.80	0.07	1.27	0.01	0.00	0.09
	Σ9	EAM	18.57	20.91	0.21	1.51	0.02	0.00	0.27
		LJ	25.85	28.06	0.14	1.41	0.01	0.00	0.18
$Ni_{0.50} Co_{0.50}$	Non GB	EAM	26.72	42.63	0.99	2.41	0.09	0.00	1.04
		LJ	34.13	62.33	1.38	2.79	0.12	0.00	1.35
	Σ7	EAM	27.32	28.49	0.07	1.27	0.01	0.00	0.09
		LJ	35.41	36.95	0.07	1.28	0.01	0.00	0.10
	Σ9	EAM	18.94	20.76	0.16	1.44	0.02	0.00	0.21
		LJ	26.82	28.67	0.11	1.36	0.01	0.00	0.15
$Ni_{0.75} Co_{0.25}$	Non GB	EAM	25.83	43.70	1.15	2.57	0.10	0.00	1.18
		LJ	33.23	60.14	1.35	2.76	0.12	0.00	1.33
	Σ7	EAM	24.98	25.99	0.07	1.27	0.01	0.00	0.09
		LJ	34.53	35.92	0.07	1.27	0.01	0.00	0.09
	Σ9	EAM	16.52	19.34	0.28	1.62	0.03	0.00	0.35
		LJ	26.17	28.16	0.13	1.38	0.01	0.00	0.16

**Figure 13:**
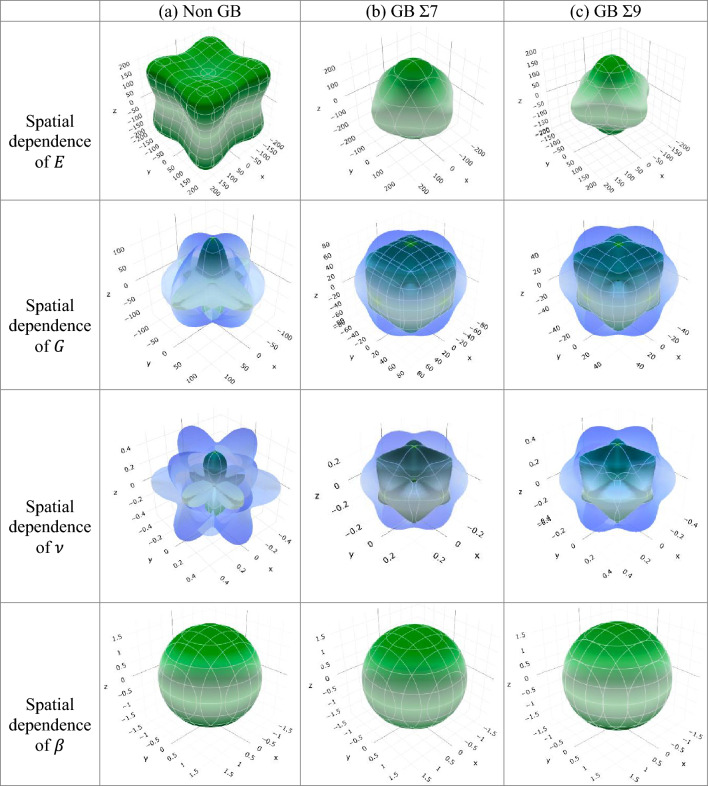
3D representation of the directional dependency of *E, G, ν* and* β* for $$NiCo$$ generated by (**a**) Non GB, (**b**) GB Σ7, and (**c**) GB Σ9 for $$ Ni_{0.50} Co_{0.50}$$ alloy composition using EAM potential.

**Figure 14:**
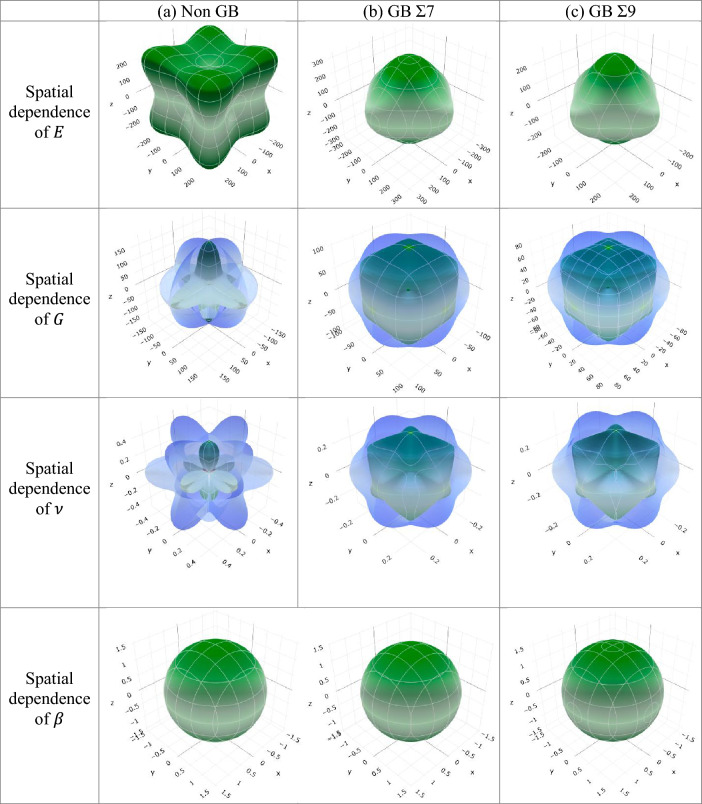
3D representation of the directional dependency of *E, G, ν* and* β* for $$NiCo$$ alloys generated by (**a**) Non GB, (**b**) GB Σ7, and (**c**) GB Σ9 for $$ Ni_{0.50} Co_{0.50}$$ alloy composition using LJ potential.

Table S7 shows the minimum and maximum limit of Young modulus, linear compressibility, shear modulus and Poisson’s ratio. It also demonstrates that the presence of grain boundaries leads to a reduction in the values of $$A_{E}$$, $$ A_{\beta }$$, $$ A_{G}$$, and $$A_{\nu }$$. It was observed that the values exhibited an increasing trend when transitioning from GB Σ7 to GB Σ9 for all compositions of alloys. The ELATE programme^80^ was used to make plots of Young’s modulus, shear modulus, Poisson’s ratio, linear compressibility, and in anisotropic 3D contours. The ELATE programme uses a matrix to show how the different alloy composition and grain boundaries affect the amounts of anisotropy in $$E$$, $$G$$, $$\nu$$ and $$\beta$$. Figures 13 and 14 show the 3D direction dependence of $$E$$, $$G$$, $$\nu$$ and $$\beta$$ for three different grain boundaries: non-GB, GB Σ7 and GB Σ9 for $$Ni_{0.50} Co_{0.50}$$ alloy composition both for EAM and LJ potentials. This shows that $$NiCo$$ alloys are not isotropic. From Figs. 13, 14 and Figs. S1–S4 the elastic moduli seem to change in different directions. The 3D spherical contour plots show clearly that isotropy is present, while the other shapes in the plots show that anisotropy is present. It has been found that $$NiCo$$ has less anisotropic qualities when grain boundaries are present, except for $$A^{B}$$.

## Conclusions

In this study, molecular dynamic simulations were used to study mechanical properties of $$NiCo$$ binary alloys with and without grain boundaries, including different alloy compositions. It is anticipated that NiCo alloys, composed of different alloy compositions, will exhibit lattice deformation in response to lattice distortions. The estimated elastic constants satisfy the Born stability criteria, ensuring mechanical stability across all investigated grain boundaries and chemical compositions. Materials with higher elastic moduli are less prone to deformation or failure in applications requiring exceptional strength and stiffness. The elastic moduli of NiCo with alloy composition $$Ni_{0.50} Co_{0.50} $$ are larger than those of others, demonstrating that it enhances their strength and hardness. Grain boundaries, notably GB Σ 9, reduce stiffness due to defects. This emphasizes the need to consider both alloy composition and grain boundary type for materials design and engineering. GB Σ7 tends to exhibit lower Poisson’s and Pugh’s ratios compared to non-grain boundary models, but these ratios increase when considering GB Σ9, which has a more pronounced impact. Alloy compositions like $$Ni_{0.75} Co_{0.25}$$ typically shows higher values for B/G and ν. The impact of the grain boundary causes the Cauchy’s pressure of $$NiCo$$ binary alloy to increase, although the impact of GB Σ9 is greater than that of GB Σ7. This value rises when the Ni content in the alloy composition is raised. The Kleinman parameter suggests that the bonding inside the material exhibits bending characteristics, resulting in a reduction in bond strength at the grain boundary for all alloy compositions. The values of the machinability index indicate that medium-entropy $$NiCo$$ alloys are highly machinable and GB Σ9 configurations with $$Ni_{0.75} Co_{0.25}$$ composition tend to exhibit higher values of the machinability. The hardness value decreases as the grain boundary type changes from non-grain to GB Σ7 and GB Σ9. But $$Ni_{0.50} Co_{0.50} $$ alloy consistently display higher hardness values. Several direction-dependent parameters indicate that $$NiCo$$ is anisotropic, while GB Σ7 reduces anisotropy. In conclusion, it is suggested that alloy composition and GB will significantly alter the mechanical properties of NiCo binary alloys.

## Supplementary Information

Supplementary Information.

## Data Availability

The data sets generated and/or analyzed in this study are available from the corresponding author on reasonable request.
