# Supplementary material for: Edge dislocations, alloy composition, and grain boundaries effects on the mechanical properties in NiCo binary alloy
Source: Sci Rep. 2024 Nov 13;14:27790. doi: 10.1038/s41598-024-65437-y (PMC11561343; doi:10.1038/s41598-024-65437-y)
Supplement: Supplementary file 1 — Supplementary Information. [file 41598_2024_65437_MOESM1_ESM.docx]

**Supporting Information:**

**Edge Dislocations, Alloy Composition, and Grain Boundaries Effects on the Mechanical Properties in** $\boldsymbol{NiCo}$ **Binary Alloy**

Md. Nadim Mahamud Nobin

E-mail: nobin160702@gmail.com

Department of Physics, Pabna University of Science and Technology, Pabna-6600, Bangladesh

Md. Lokman Ali^*^

Corresponding author’s E-mail: lokman.cu12@gmail.com

Department of Physics, Pabna University of Science and Technology, Pabna-6600, Bangladesh

Md. Khairul Alam

E-mail: drmkalam14@gmail.com

Department of Physics, Pabna University of Science and Technology, Pabna-6600, Bangladesh

**Lattice distortion, yield strength, MSAD and CRSS**

Table S1: MD simulated Lattice constant, MSAD, CRSS and yield strength of $NiCo$ binary alloys.

| Alloy composition | lattice constant | MSAD  $\boldsymbol{Pm}^{\boldsymbol{2}}$ | $\sqrt{\boldsymbol{MSAD}}$  $\boldsymbol{Pm}$ | CRSS | Yield strength |
| --- | --- | --- | --- | --- | --- |
| $\boldsymbol{Ni}_{\boldsymbol{0.25}}\boldsymbol{Co}_{\boldsymbol{0.75}}$ | 3.42 | 16.36 | 4.04 | 1.05 | 482.07 |
| $\boldsymbol{Ni}_{\boldsymbol{0.50}}\boldsymbol{Co}_{\boldsymbol{0.50}}$ | 3.37 | 25.18 | 5.02 | 0.80 | 611.07 |
| $\boldsymbol{Ni}_{\boldsymbol{0.75}}\boldsymbol{Co}_{\boldsymbol{0.25}}$ | 3.33 | 12.47 | 3.53 | 0.51 | 421.50 |

**Elastic stiffness constant**

Table S2: Calculated elastic constants of ${Ni}_{0.25}{Co}_{0.75}$ alloys.

| Grain boundary type | Potential | *C*_11_ | *C*_12_ | *C*_44_ | *C*_12_-*C*_44_ |
| --- | --- | --- | --- | --- | --- |
| Non-grain boundary | EAM | 254.30 | 156.40 | 126.27 | 30.13 |
|  | LJ | 298.59 | 175.42 | 170.22 | 5.2 |
| Σ7 | EAM | 305.59 | 133.29 | 70.67 | 62.62 |
|  | LJ | 382.89 | 143.23 | 94.36 | 48.87 |
| Σ9 | EAM | 278.47 | 132.64 | 48.15 | 84.49 |
|  | LJ | 337.64 | 144.50 | 68.56 | 75.94 |

Table S3: Calculated elastic constants of ${Ni}_{0.50}{Co}_{0.50}$ alloys.

| Grain boundary type | Potential | *C*_11_ | *C*_1 2_ | *C*_44_ | *C*_12_-*C*_44_ |
| --- | --- | --- | --- | --- | --- |
| Non-grain boundary | EAM | 259.96 | 155.98 | 125.44 | 30.54 |
|  | LJ | 309.30 | 183.33 | 175.66 | 7.67 |
| Σ7 | EAM | 320.72 | 129.77 | 74.89 | 54.88 |
|  | LJ | 398.07 | 150.16 | 96.97 | 53.19 |
| Σ9 | EAM | 279.58 | 136.03 | 49.89 | 86.14 |
|  | LJ | 348.03 | 152.26 | 71.93 | 80.33 |

Table S4: Calculated elastic constants of ${Ni}_{0.75}{Co}_{0.25}$ alloys.

| Grain boundary type | Potential | *C*_11_ | *C*_12_ | *C*_44_ | *C*_12_-*C*_44_ |
| --- | --- | --- | --- | --- | --- |
| Non-grain boundary | EAM | 253.88 | 155.75 | 126.22 | 29.53 |
|  | LJ | 298.39 | 175.34 | 169.98 | 5.36 |
| Σ7 | EAM | 306.88 | 133.03 | 68.65 | 64.38 |
|  | LJ | 383.55 | 143.31 | 94.90 | 48.41 |
| Σ9 | EAM | 272.97 | 136.98 | 41.98 | 95.00 |
|  | LJ | 338.03 | 145.00 | 69.84 | 75.16 |

**Mechanical properties**

The elastic moduli of $NiCo$ binary alloys are commonly determined through the application of the Voigt-Reuss-Hill (VRH) averaging procedures, utilizing the following equation [1].

Using the VRH method, the bulk and shear moduli of a cubic system are [2]:

| *B =* $\frac{1}{2}$ *(*$B_{R}+B_{v})$ | (1) |
| --- | --- |
| *G =* $\frac{1}{2}$ *(*$G_{\nu}+G_{R})$ | (2) |

Using the following equations the bulk and shear moduli of Voigt ($B_{v}$and $G_{\nu}$, respectively) and the bulk and shear moduli of Reuss ($B_{R}$and $G_{R}$) are calculated as follows [3]:

| $B_{\nu}=B_{R}= \frac{{(C}_{11}+2C_{12})}{3}$ | (3) |
| --- | --- |
| $G_{\nu}=\frac{(C_{11}-C_{12}+3C_{44})}{5}$ | (4) |
| $G_{R} = \frac{{5C}_{44}\left( C_{11}-C_{12} \right)}{[{4C}_{44}+3(C_{11}-C_{12}]}$ | (5) |

The following equations can be used to calculate, the Young’s modulus (*E*), Poisson’s ratio ($\nu$) and anisotropy factor (*A*) [4]:

| $E=\frac{9GB}{3B + G}$ | (6) |
| --- | --- |
| $\nu=\frac{3B - 2G}{2(3B + G)}$ | (7) |
| $A =\frac{2C_{44}}{{(C}_{11}-C_{12})}$ | (8) |

The equation used to calculate the Kleinman constant is given by the following expression [5]:

| $\zeta=\frac{C_{11}+8C_{12}}{7C_{11}+2C_{12}}$ | (9) |
| --- | --- |

The machinability index, $\mu_{M}$=*B/*$C_{44}$,, is a critical efficiency parameter for engineering’s long-term uses. The value of $\mu_{M}$ is used to characterize the slicability of a solid. Adjustments and dry lubrication are simplified at high $\mu_{M}$ [6].

$$\mu_{M}=\frac{B_{H}}{C_{44}}$$

Table S5: The effect of alloy composition and grain boundaries on *B* (GPa), *G* (GPa), *E* (GPa), *G/B*,$\mu_{M}$, *ζ*, and 𝜈 of $NiCo$binary alloys.

| Alloy Composition | Grain boundary type | *B* | | | | *G* | | | $\frac{\boldsymbol{B}_{\boldsymbol{V}}}{\boldsymbol{B}_{\boldsymbol{R}}}$ | $\frac{\boldsymbol{G}_{\boldsymbol{V}}}{\boldsymbol{G}_{\boldsymbol{R}}}$ | $\frac{\boldsymbol{G}}{\boldsymbol{B}}$ | $\boldsymbol{\mu}_{\boldsymbol{M}}$ | $\boldsymbol{\zeta}$ |
| --- | --- | --- | --- | --- | --- | --- | --- | --- | --- | --- | --- | --- | --- |
|  |  | $B_{V}$ | $B_{R}$ | | $B_{H}$ | $G_{V}$ | $G_{R}$ | $G_{H}$ |  |  |  |  |  |
| $\boldsymbol{Ni}_{\boldsymbol{0.25}}\boldsymbol{Co}_{\boldsymbol{0.75}}$ | Non GB | 189.03 | | 189.03 | 189.03 | 95.34 | 77.38 | 86.36 | 1 | 1.23 | 0.46 | 1.50 | 0.72 |
|  | Σ7 | 190.72 | 190.72 | | 190.72 | 76.86 | 76.14 | 76.50 | 1 | 1.01 | 0.40 | 2.70 | 0.57 |
|  | Σ9 | 181.25 | 181.25 | | 181.25 | 58.05 | 55.72 | 56.88 | 1 | 1.04 | 0.31 | 3.76 | 0.60 |
| $\boldsymbol{Ni}_{\boldsymbol{0.50}}\boldsymbol{Co}_{\boldsymbol{0.50}}$ | Non GB | 190.64 | 190.64 | | 190.64 | 96.06 | 80.15 | 88.10 | 1 | 1.20 | 0.46 | 1.52 | 0.71 |
|  | Σ7 | 193.42 | 193.42 | | 193.42 | 83.12 | 81.96 | 82.54 | 1 | 1.01 | 0.43 | 2.58 | 0.54 |
|  | Σ9 | 183.88 | 183.88 | | 183.88 | 58.65 | 56.82 | 57.73 | 1 | 1.03 | 0.31 | 3.69 | 0.61 |
| $\boldsymbol{Ni}_{\boldsymbol{0.75}}\boldsymbol{Co}_{\boldsymbol{0.25}}$ | Non GB | 188.46 | 188.46 | | 188.46 | 95.36 | 77.48 | 86.42 | 1 | 1.23 | 0.46 | 1.49 | 0.72 |
|  | Σ7 | 190.98 | 190.98 | | 190.98 | 75.96 | 74.95 | 75.46 | 1 | 1.01 | 0.40 | 2.78 | 0.70 |
|  | Σ9 | 182.31 | 182.31 | | 182.31 | 52.39 | 49.57 | 50.98 | 1 | 1.06 | 0.28 | 4.34 | 0.57 |

Table S6: The effect of alloy composition and grain boundaries on *B* (GPa), *G* (GPa), *E* (GPa), *G/B*,$\mu_{M}$, *ζ*, and 𝜈 of $NiCo$alloys.

| Alloy Composition | Grain boundary type | *B* | | | | *G* | | | | $\frac{\boldsymbol{B}_{\boldsymbol{V}}}{\boldsymbol{B}_{\boldsymbol{R}}}$ | $\frac{\boldsymbol{G}_{\boldsymbol{V}}}{\boldsymbol{G}_{\boldsymbol{R}}}$ | $\frac{\boldsymbol{G}}{\boldsymbol{B}}$ | $\boldsymbol{\mu}_{\boldsymbol{M}}$ | $\boldsymbol{\zeta}$ |
| --- | --- | --- | --- | --- | --- | --- | --- | --- | --- | --- | --- | --- | --- | --- |
|  |  | $B_{V}$ | | $B_{R}$ | $B_{H}$ | $G_{V}$ | | $G_{R}$ | $G_{H}$ |  |  |  |  |  |
| $\boldsymbol{Ni}_{\boldsymbol{0.25}}\boldsymbol{Co}_{\boldsymbol{0.75}}$ | Non GB | 216.48 | 216.48 | | 216.48 | | 126.77 | 99.80 | 113.28 | 1 | 1.27 | 0.52 | 1.27 | 0.70 |
|  | Σ7 | 223.12 | | 223.12 | 223.12 | | 104.55 | 103.13 | 103.84 | 1 | 1.01 | 0.47 | 2.36 | 0.52 |
|  | Σ9 | 208.88 | | 208.88 | 208.88 | | 79.76 | 77.56 | 78.66 | 1 | 1.03 | 0.38 | 3.05 | 0.56 |
| $\boldsymbol{Ni}_{\boldsymbol{0.50}}\boldsymbol{Co}_{\boldsymbol{0.50}}$ | Non GB | 225.32 | | 225.32 | 225.32 | | 130.59 | 102.39 | 116.49 | 1 | 1.28 | 0.52 | 1.28 | 0.70 |
|  | Σ7 | 232.80 | | 232.80 | 232.80 | | 107.76 | 106.22 | 106.99 | 1 | 1.01 | 0.46 | 2.40 | 0.52 |
|  | Σ9 | 217.52 | | 217.52 | 217.52 | | 82.31 | 80.46 | 81.39 | 1 | 1.02 | 0.37 | 3.02 | 0.57 |
| $\boldsymbol{Ni}_{\boldsymbol{0.75}}\boldsymbol{Co}_{\boldsymbol{0.25}}$ | Non GB | 216.36 | | 216.36 | 216.36 | | 126.60 | 99.69 | 113.14 | 1 | 1.27 | 0.52 | 1.27 | 0.70 |
|  | Σ7 | 223.39 | | 223.39 | 223.39 | | 104.99 | 130.60 | 104.29 | 1 | 0.80 | 0.47 | 2.35 | 0.57 |
|  | Σ9 | 209.34 | | 209.34 | 209.34 | | 80.51 | 78.52 | 79.52 | 1 | 1.03 | 0.38 | 3.00 | 0.51 |

**Hardness calculation**

The determination of hardness involves the utilization of semi-empirical correlations that exist between Vickers hardness ($H_{v}$) and the macroscopic variables used in hardness prediction (*E*, *G*, *B*, $\nu$, and *G/B*) [7].

| $H_{1}= 0.0963B$ | (10) |
| --- | --- |
| $H_{2}=0.0607E$ | (11) |
| $H_{3}$ $= 0.1475G$ | (12) |
| $H_{4}= 0.0635E$ | (13) |
| $H_{5}$ $= -2.899+ 0.1769G$ | (14) |
| $H_{6}=\frac{\left( 1-2\nu\right)B}{6(1+\nu)}$ | (15) |
| $H_{7}=\frac{\left( 1-2\nu\right)E}{6(1+\nu)}$ | (16) |

**Direction dependent Anisotropic property**

The formula for determining the universal anisotropy factor, $A^{U}$ is as follows: [8]:

$$A^{U}=5\frac{G_{V}}{G_{R}}+\frac{B_{V}}{B_{R}}-6\geq0$$

If the value of $A^{U}$ is zero, the material is an isotropic crystal, whereas any other number indicates that it is anisotropic. The bulk anisotropy$A^{B}$ and the shear anisotropy $A^{G}$are two further types of anisotropy indices that may be computed with the help of the following equations

If $A^{U}$ = 0, then the material is isotropic, and if it is not, then the material is anisotropic. There are several different kinds of anisotropy indices, and the following formulae can be used to determine the bulk anisotropy $A^{B}$ and the shear anisotropy $A^{G}$ [9, 10]:

$$A^{G}=\frac{G_{V}-G_{R}}{2G_{H}}$$

The shear anisotropy factor can be used to describe the dissimilarity between atomic bonds in different crystallographic planes [10-13]. The equivalent Zener anisotropy factor, denoted by $A^{eq}$, is written as follows: [10]:

$$A^{eq}=\left( 1+\frac{5}{12}A^{U} \right)+\sqrt{\left( 1+\frac{5}{12}A^{U} \right)^{2}-1}$$

The following formula can be used to determine the universal Log-Euclidean index using the log Euclidean method [10, 14]:

$$A^{L} =\sqrt{[\ln\left( \frac{B_{V}}{B_{R}} \right)+5[\ln(\frac{C_{44}^{V}}{C_{44}^{R}})]^{2}}$$

For the Voigt and the Reuss, the corresponding $C_{44}$ values can be written as:

$$C_{44}^{R}=\frac{5}{3} \frac{C_{44}(C_{11}-C_{12})}{3\left( C_{11}- C_{12} \right)+4C_{44}}$$

$C_{44}^{V}$*=*$\frac{3}{5}\frac{(C_{11}-C_{12}-2C_{44})^{2}}{3\left( C_{11}- C_{12} \right)+4C_{44}}$

Table S7**.** Calculated minimum and maximum value of elastic moduli of $NiCo$ alloys.

| Alloy Composition | Grain boundary type | Potential | Young’s modulus | | | | Liner compressibility | | | | Shear modulus | | | Poisson’s ratio | | |
| --- | --- | --- | --- | --- | --- | --- | --- | --- | --- | --- | --- | --- | --- | --- | --- | --- |
|  |  |  | $E_{min}$ | $E_{max}$ | | $A_{E}$ | $\beta_{min}$ | $\beta_{max}$ | | $A_{\beta}$ | $G_{min}$ | $G_{max}$ | $A_{G}$ | $\nu_{min}$ | $\nu_{max}$ | $A_{\nu}$ |
| $\boldsymbol{Ni}_{\boldsymbol{0.25}}\boldsymbol{Co}_{\boldsymbol{0.75}}$ | Non GB | EAM | 135.18 | 309.83 | 2.29 | | 1.76 | | 1.76 | 1.00 | 48.95 | 126.27 | 2.58 | -0.073 | 0.66 | ∞ |
|  |  | LJ | 168.75 | 404.61 | 2.39 | | 1.54 | | 1.54 | 1.00 | 61.59 | 170.22 | 2.76 | -0.12 | 0.66 | ∞ |
|  | Σ7 | EAM | 188.70 | 224.63 | 1.19 | | 1.75 | | 1.75 | 1.00 | 70.67 | 86.15 | 1.22 | 0.27 | 0.39 | 1.47 |
|  |  | LJ | 248.10 | 304.90 | 1.23 | | 1.49 | | 1.49 | 1.00 | 94.36 | 119.83 | 1.27 | 0.23 | 0.38 | 1.63 |
|  | Σ9 | EAM | 132.69 | 192.88 | 1.45 | | 1.84 | | 1.84 | 1.00 | 48.15 | 72.91 | 1.51 | 0.24 | 0.49 | 2.05 |
|  |  | LJ | 185.40 | 251.03 | 1.35 | | 1.59 | | 1.59 | 1.00 | 68.56 | 96.57 | 1.41 | 0.24 | 0.45 | 1.89 |
| $\boldsymbol{Ni}_{\boldsymbol{0.50}}\boldsymbol{Co}_{\boldsymbol{0.50}}$ | Non GB | EAM | 142.98 | 308.62 | 2.16 | | 1.75 | | 1.75 | 1.00 | 51.99 | 125.44 | 2.41 | -0.05 | 0.63 | ∞ |
|  |  | LJ | 172.86 | 418.28 | 2.42 | | 1.48 | | 1.48 | 1.00 | 62.99 | 175.66 | 2.79 | -0.12 | 0.66 | ∞ |
|  | Σ7 | EAM | 198.98 | 245.96 | 1.24 | | 1.72 | | 1.72 | 1.00 | 74.89 | 95.48 | 1.28 | 0.24 | 0.39 | 1.61 |
|  |  | LJ | 255.44 | 315.81 | 1.24 | | 1.43 | | 1.43 | 1.00 | 96.97 | 123.95 | 1.28 | 0.23 | 0.38 | 1.65 |
|  | Σ9 | EAM | 137.26 | 190.54 | 1.39 | | 1.81 | | 1.81 | 1.00 | 49.89 | 71.78 | 1.44 | 0.25 | 0.48 | 1.89 |
|  |  | LJ | 194.37 | 255.35 | 1.31 | | 1.53 | | 1.53 | 1.00 | 71.93 | 97.89 | 1.36 | 0.25 | 0.44 | 1.77 |
| $\boldsymbol{Ni}_{\boldsymbol{0.75}}\boldsymbol{Co}_{\boldsymbol{0.25}}$ | Non GB | EAM | 135.44 | 309.55 | 2.29 | | 1.77 | | 1.77 | 1.00 | 49.07 | 126.22 | 2.57 | -0.07 | 0.66 | ∞ |
|  |  | LJ | 168.59 | 404.11 | 2.39 | | 1.54 | | 1.54 | 1.00 | 61.53 | 169.98 | 2.76 | -0.12 | 0.66 | ∞ |
|  | Σ7 | EAM | 183.91 | 226.42 | 1.23 | | 1.75 | | 1.75 | 1.00 | 68.65 | 86.93 | 1.27 | 0.26 | 0.41 | 1.57 |
|  |  | LJ | 249.39 | 305.59 | 1.23 | | 1.49 | | 1.49 | 1.00 | 94.90 | 120.12 | 1.27 | 0.23 | 0.38 | 1.62 |
|  | Σ9 | EAM | 116.96 | 181.43 | 1.55 | | 1.83 | | 1.83 | 1.00 | 41.98 | 67.99 | 1.62 | 0.24 | 0.53 | 2.24 |
|  |  | LJ | 188.55 | 250.98 | 1.33 | | 1.59 | | 1.59 | 1.00 | 69.84 | 96.52 | 1.38 | 0.24 | 0.44 | 1.83 |

|  | (a) Non GB | (b) GB Σ7 | (c) GB Σ9 |
| --- | --- | --- | --- |
| Spatial dependence of $E$ | 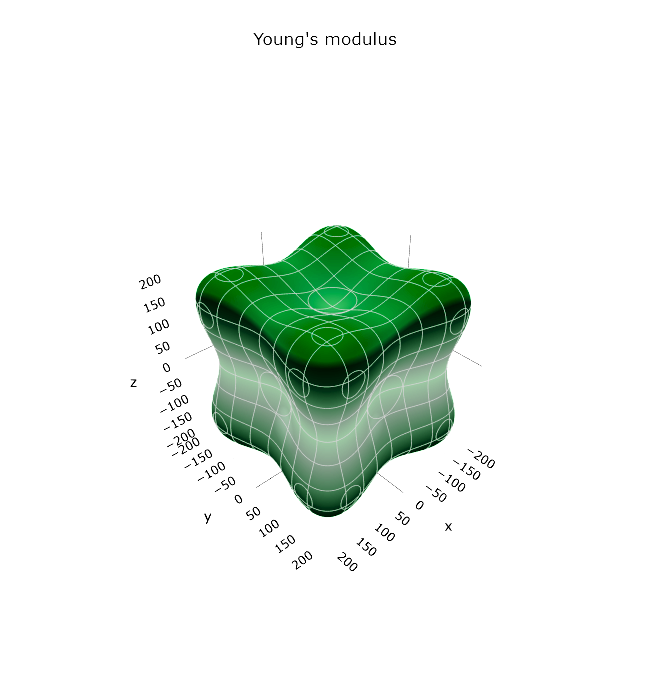 | 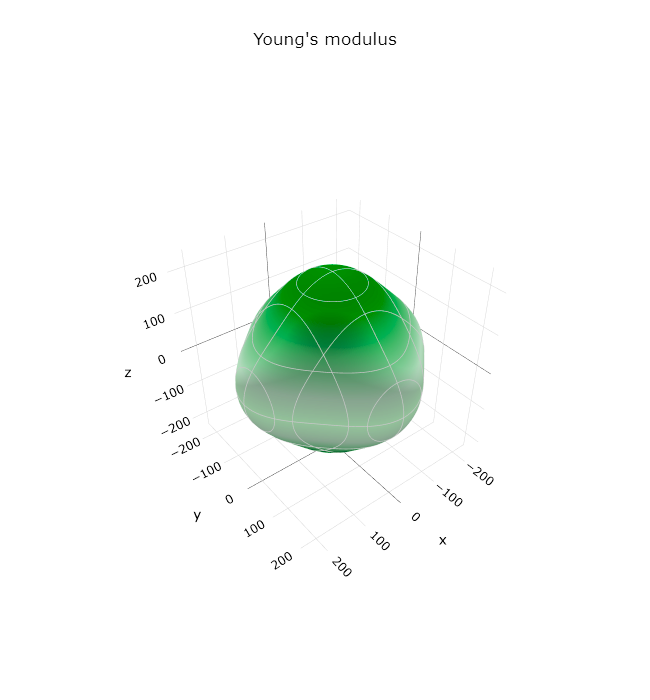 | 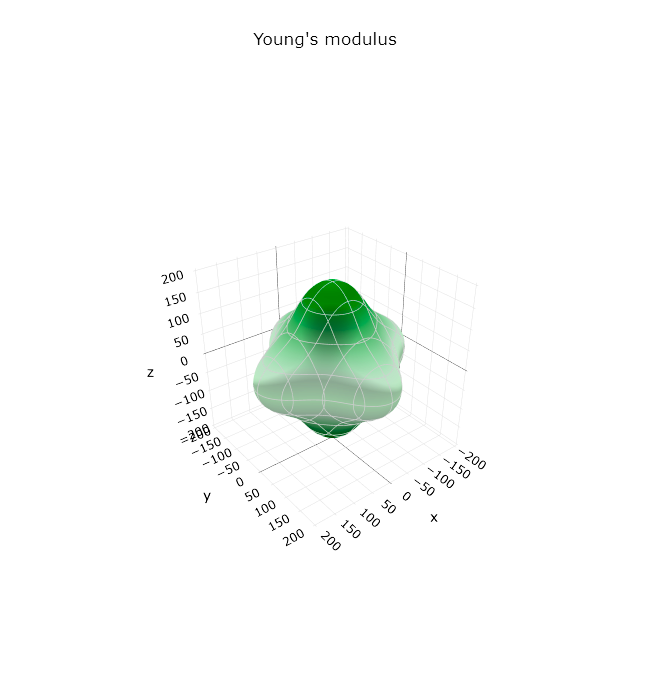 |
| Spatial dependence of $G$ | 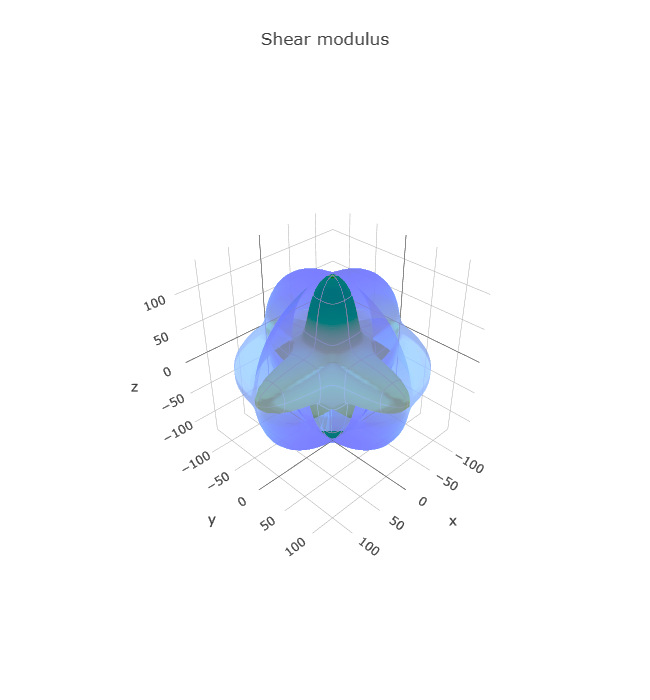 | 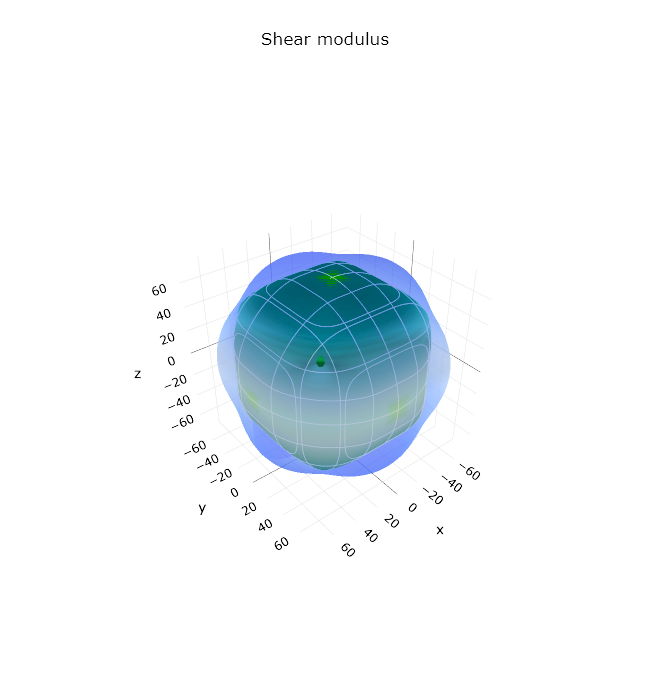 | 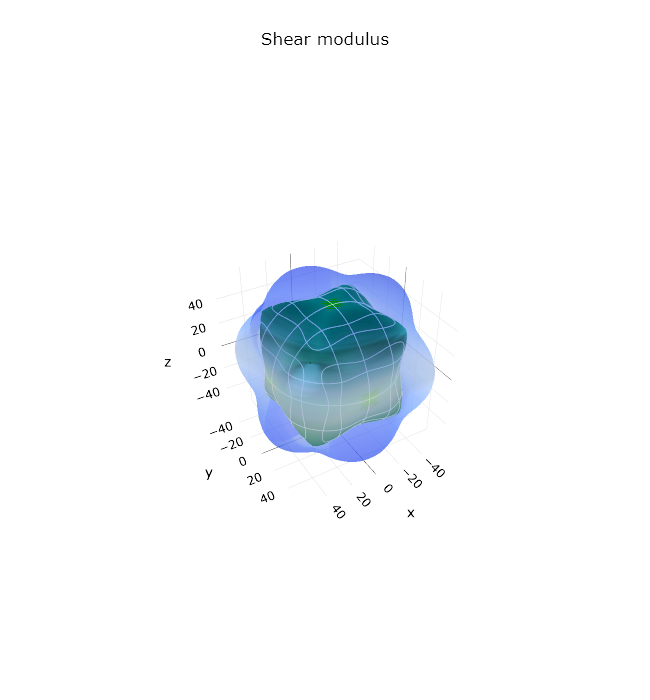 |
| Spatial dependence of $\nu$ | 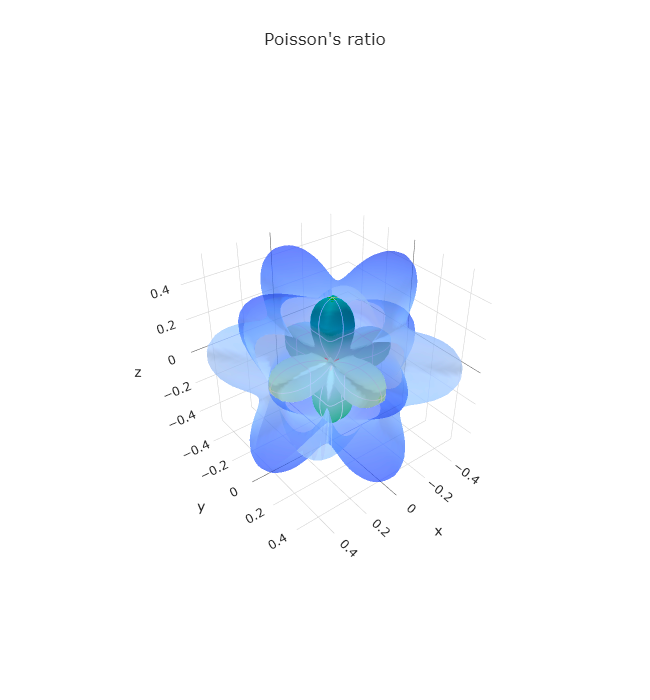 | 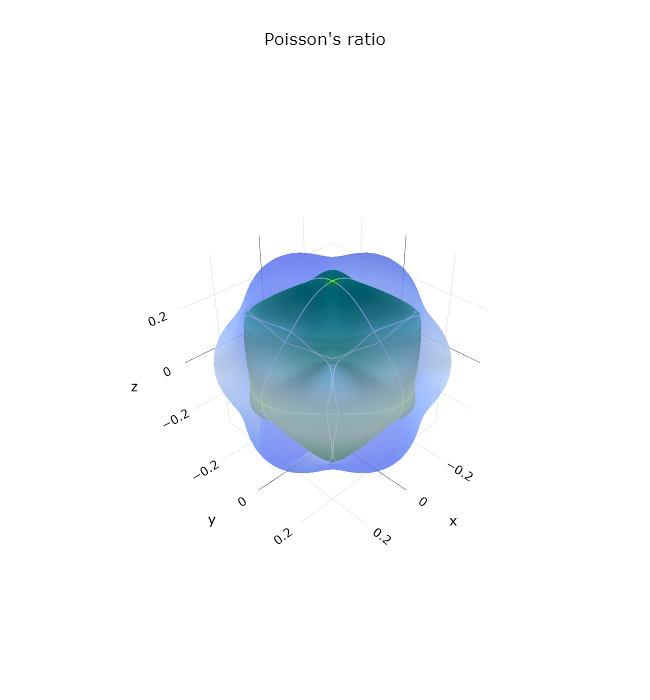 | 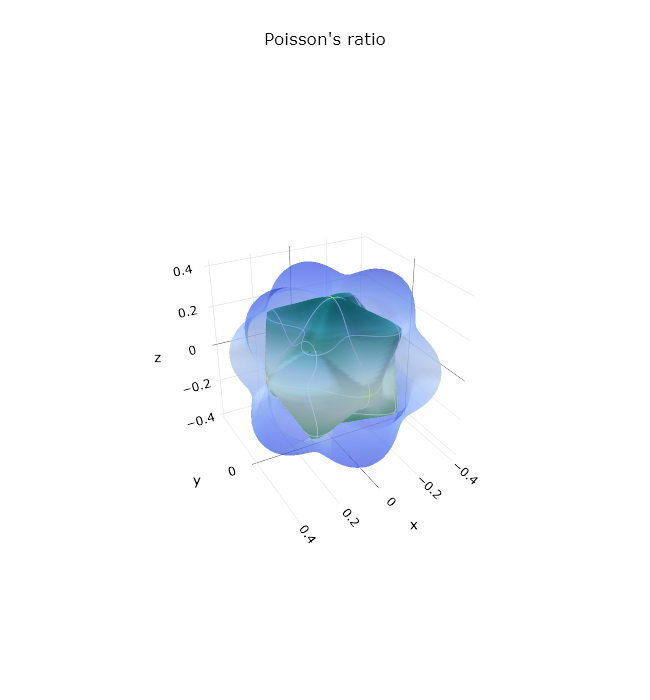 |
| Spatial dependence of $\beta$ | 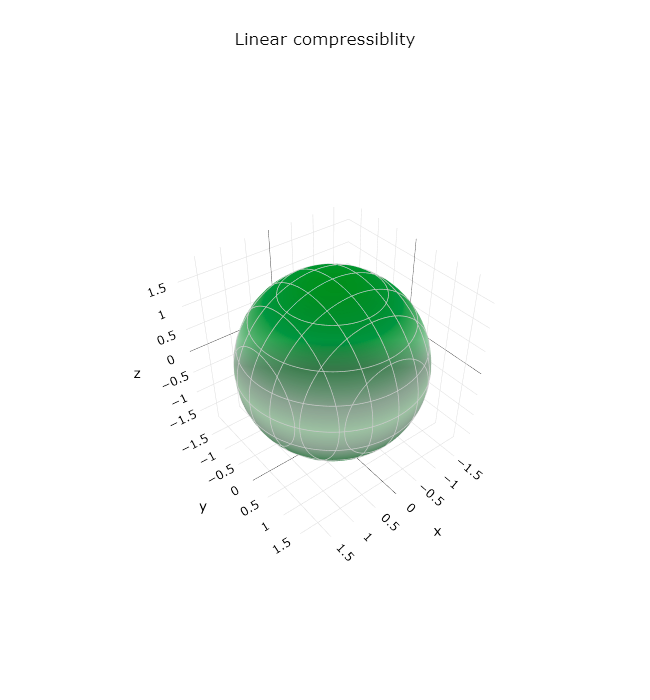 | 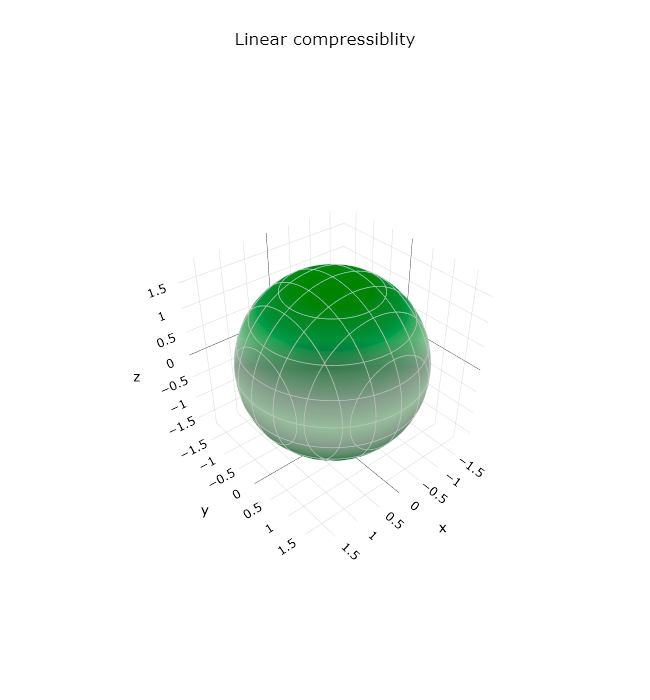 | 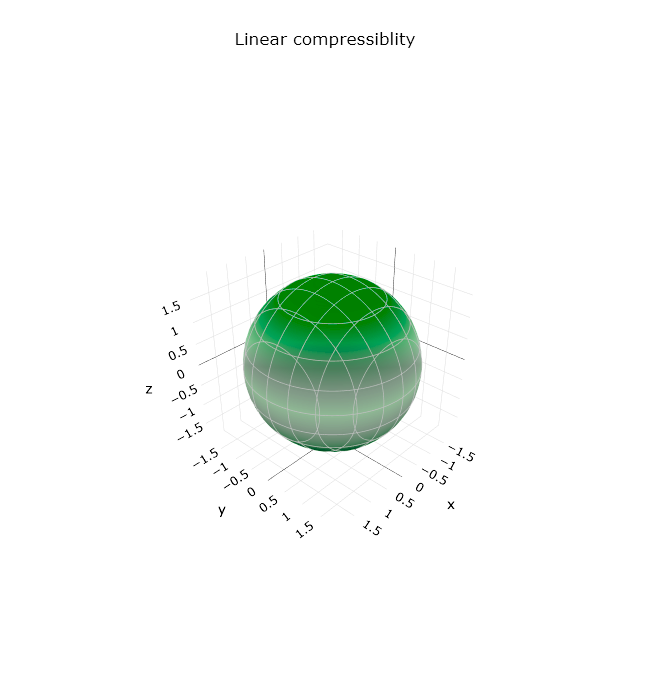 |

**Figure S1:** 3D representation of the directional dependency of *E, G, ν and β* for $NiCo$ binary alloys generated by (a) Non GB, (b) GB Σ7, and (c) GB Σ9 for ${Ni}_{0.25}{Co}_{0.75}$ alloys.

|  | (a) Non GB | (b) GB Σ7 | (c) GB Σ9 |
| --- | --- | --- | --- |
| Spatial dependence of $E$ | 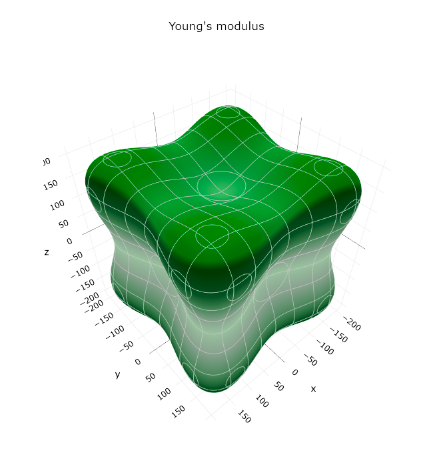 | 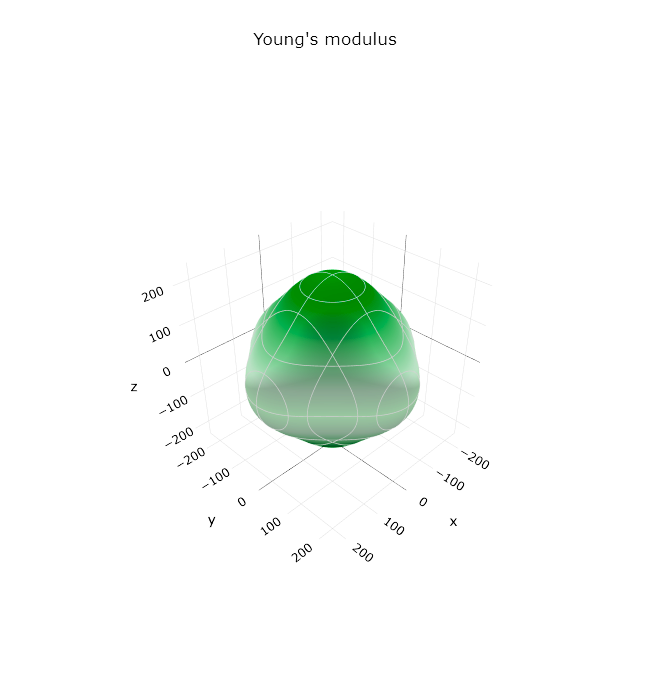 | 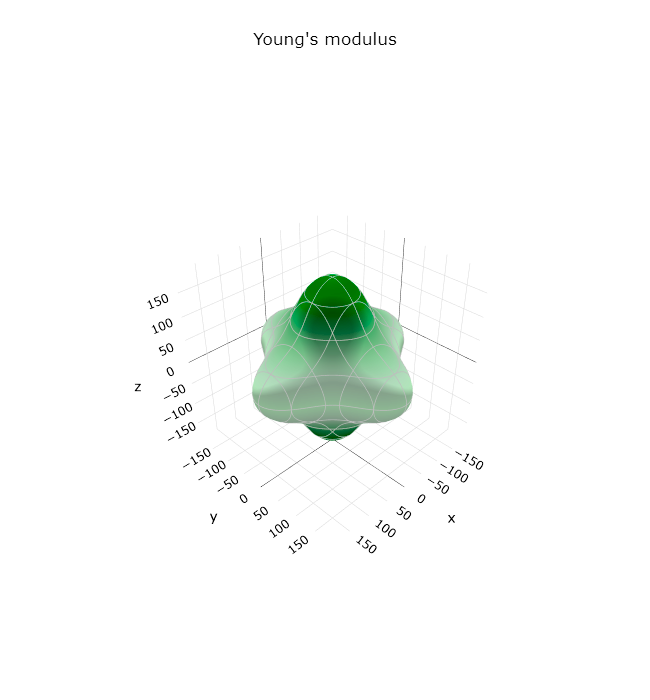 |
| Spatial dependence of $G$ | 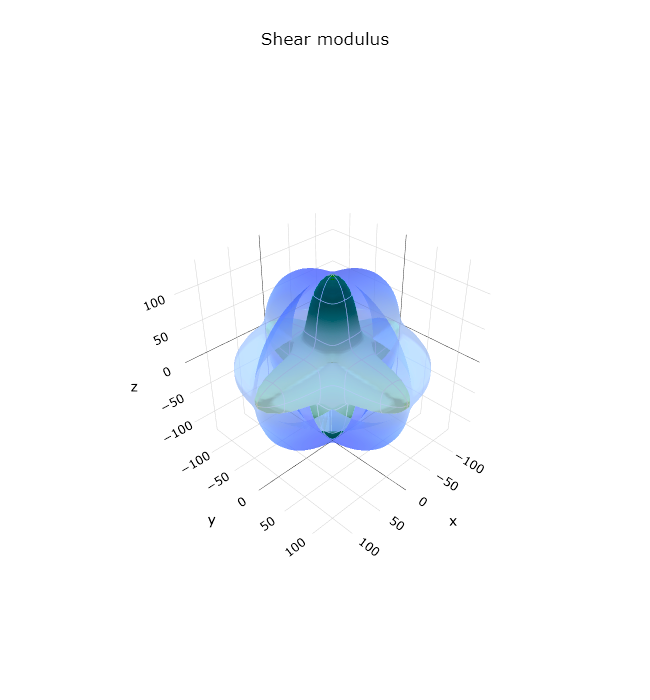 | 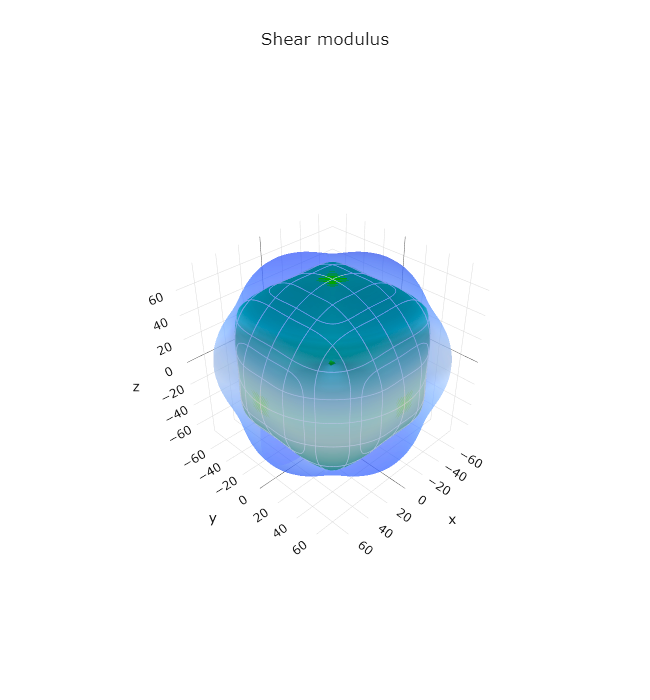 | 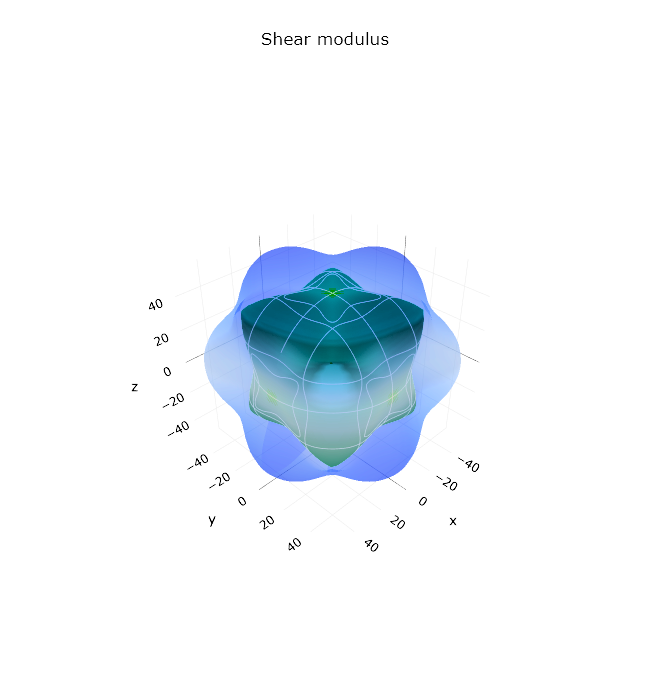 |
| Spatial dependence of $\nu$ | 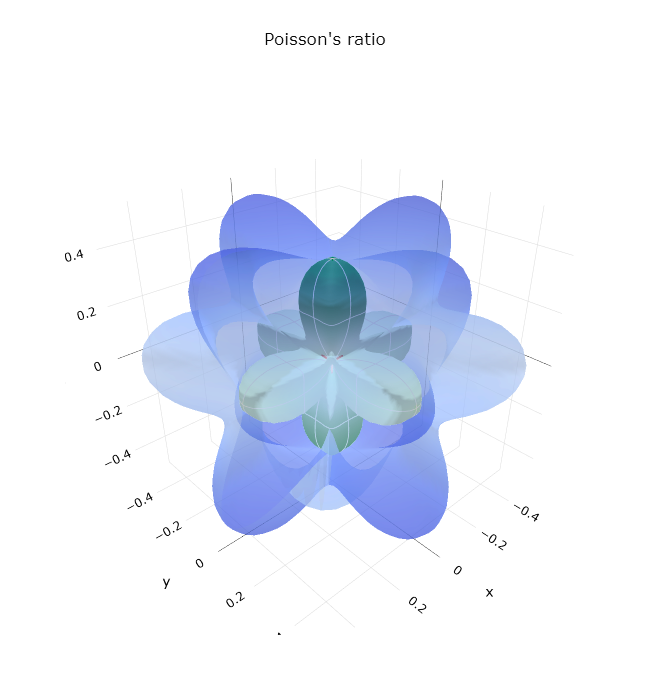 | 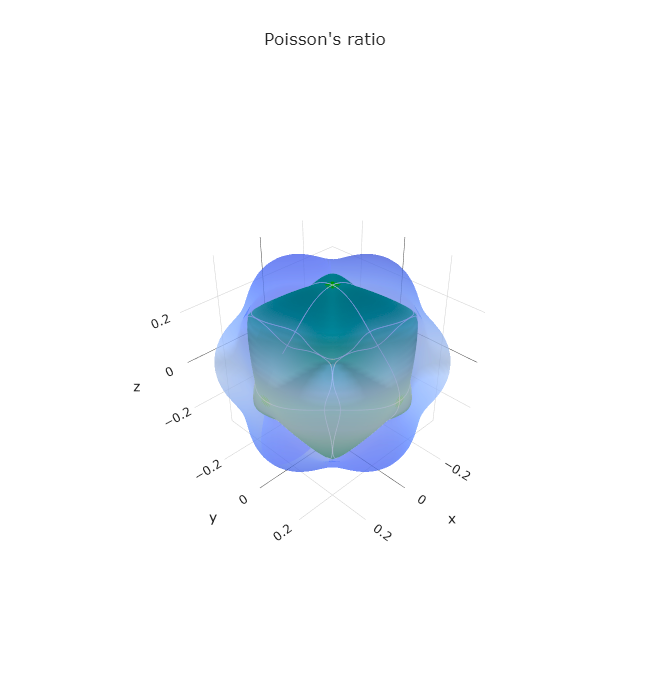 | 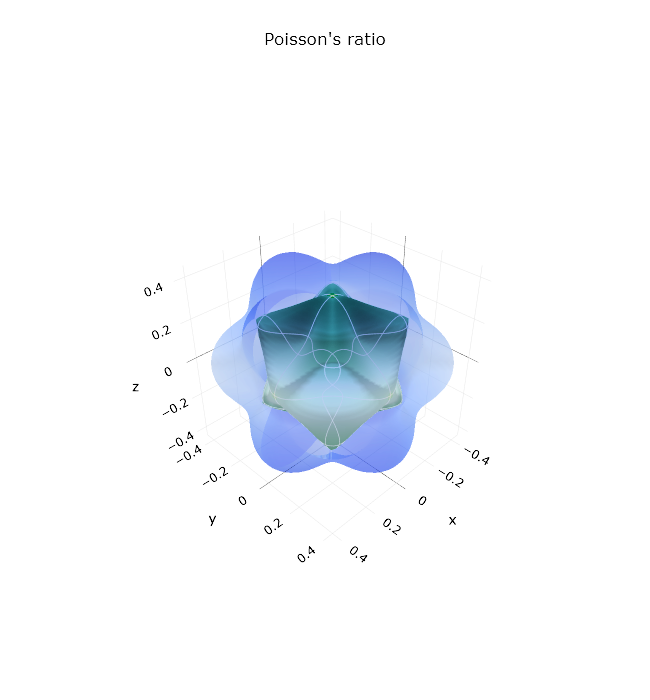 |
| Spatial dependence of $\beta$ | 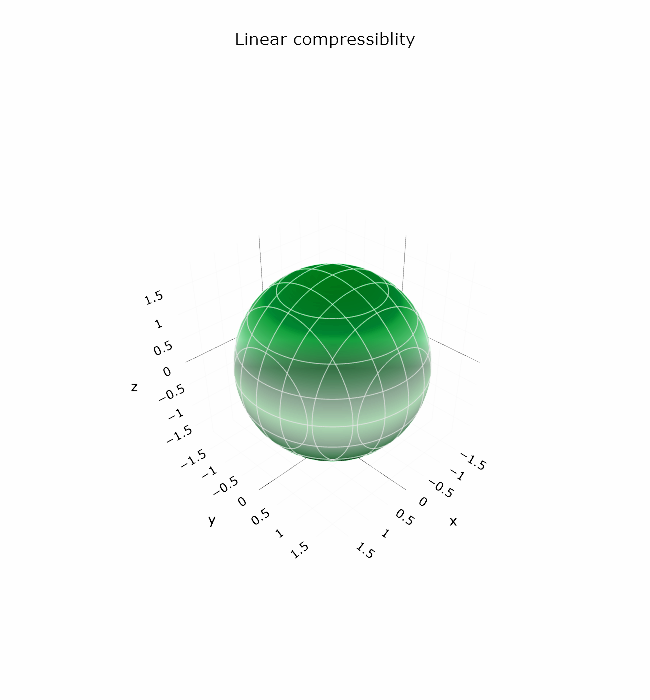 | 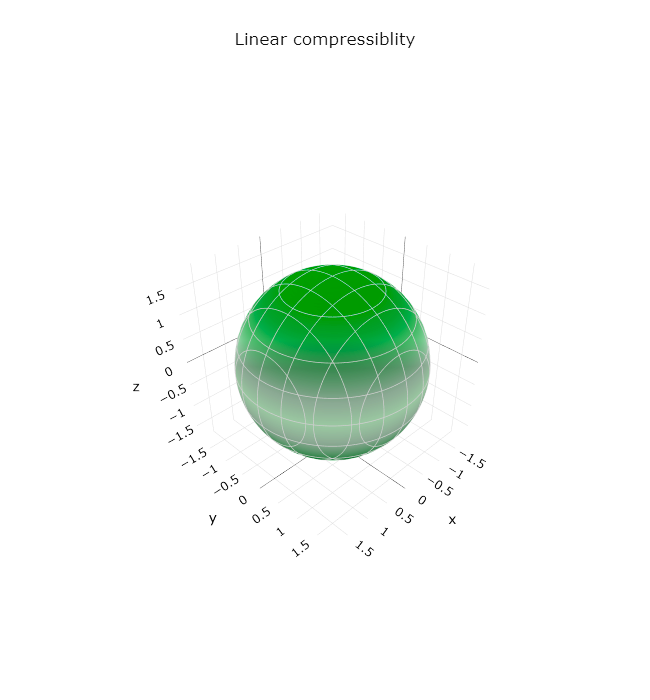 | 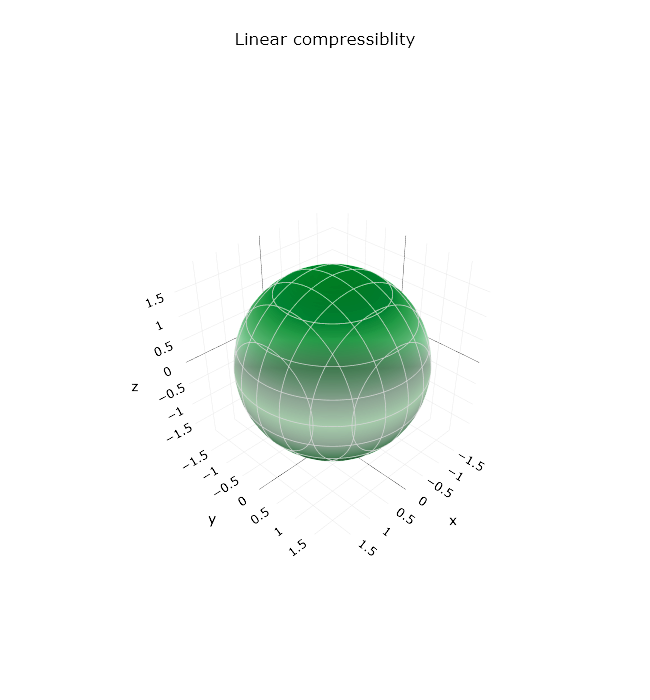 |

**Figure S2:** 3D representation of the directional dependency of *E, G, ν and β* for $NiCo$ binary alloys generated by (a) Non GB, (b) GB Σ7, and (c) GB Σ9 for ${Ni}_{0.75}{Co}_{0.25}$ alloys.

|  | (a) Non GB | (b) GB Σ7 | (c) GB Σ9 |
| --- | --- | --- | --- |
| Spatial dependence of $E$ | 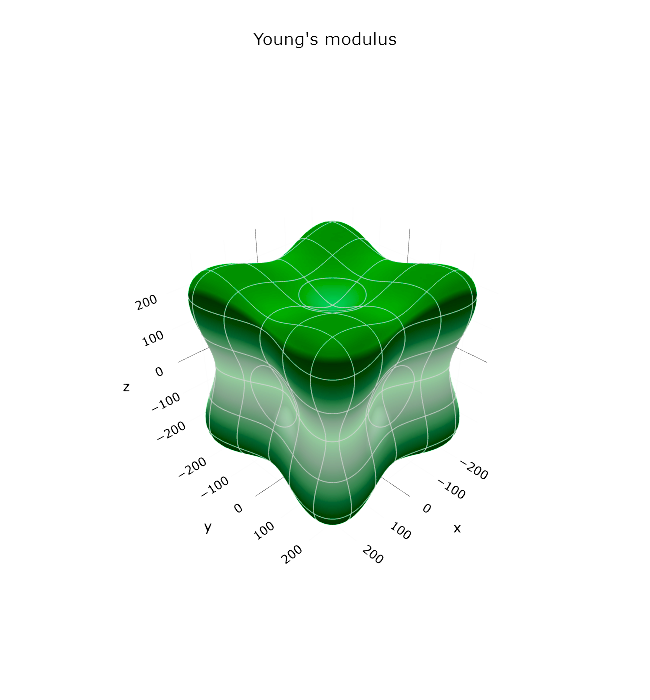 | 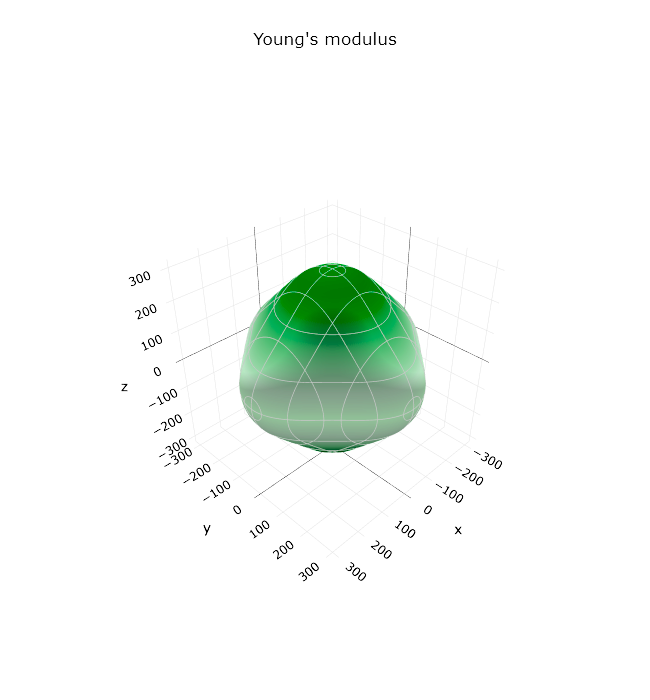 | 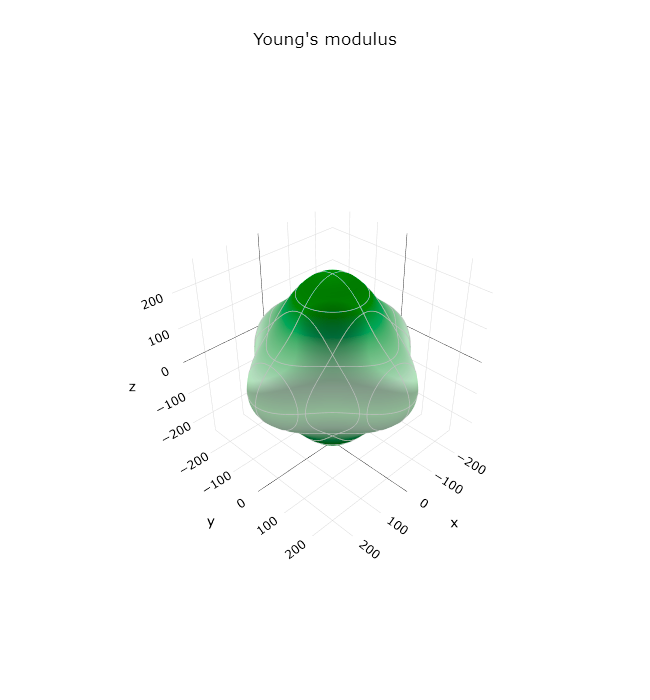 |
| Spatial dependence of $G$ | 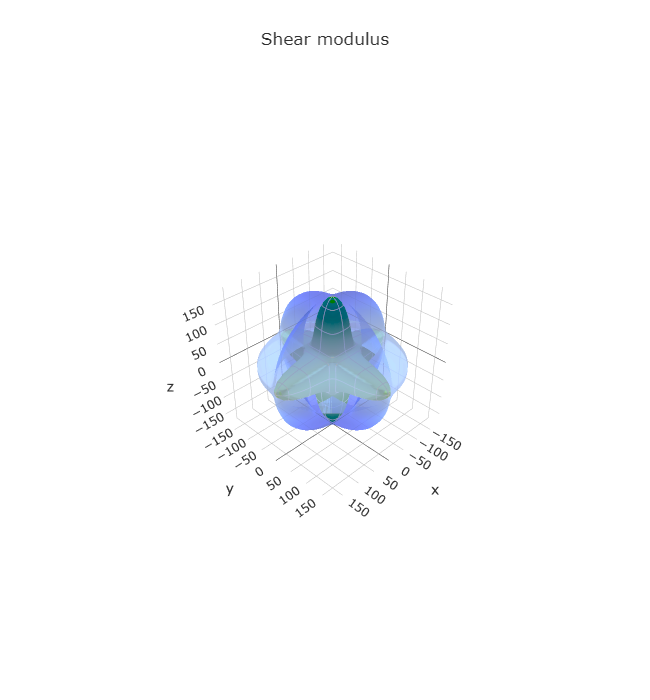 | 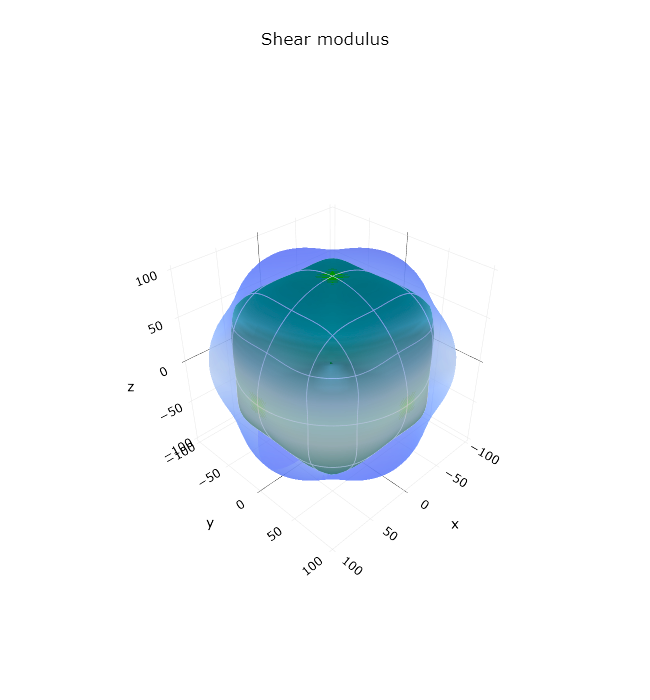 | 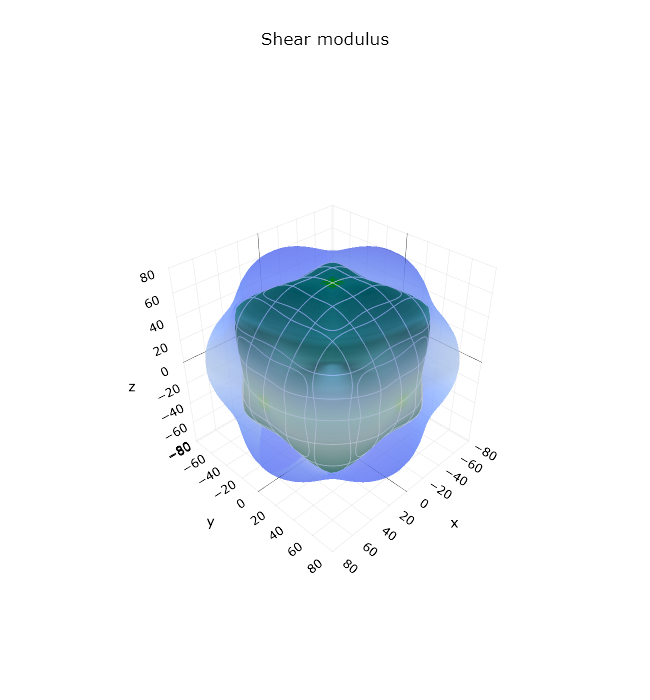 |
| Spatial dependence of $\nu$ | 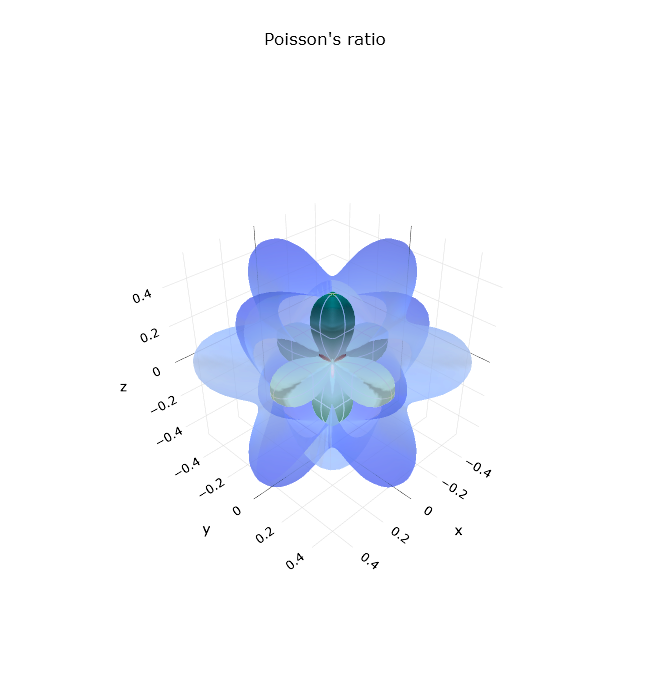 | 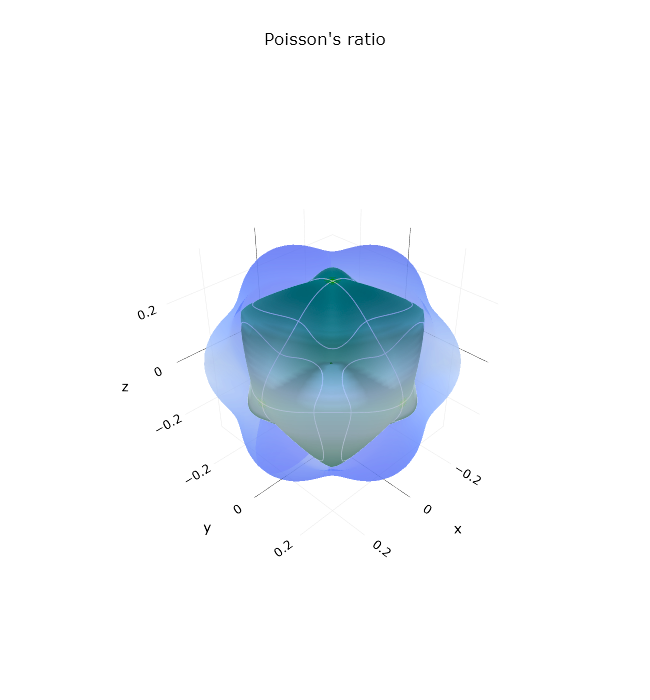 | 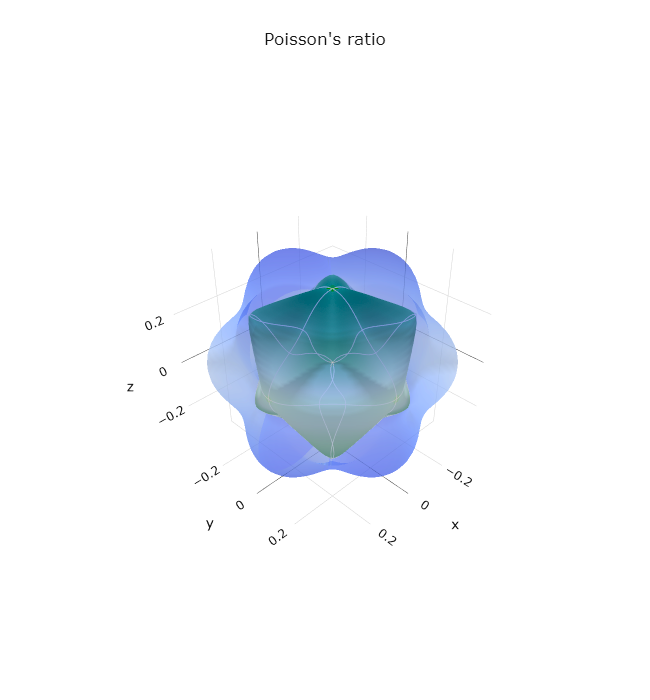 |
| Spatial dependence of $\beta$ | 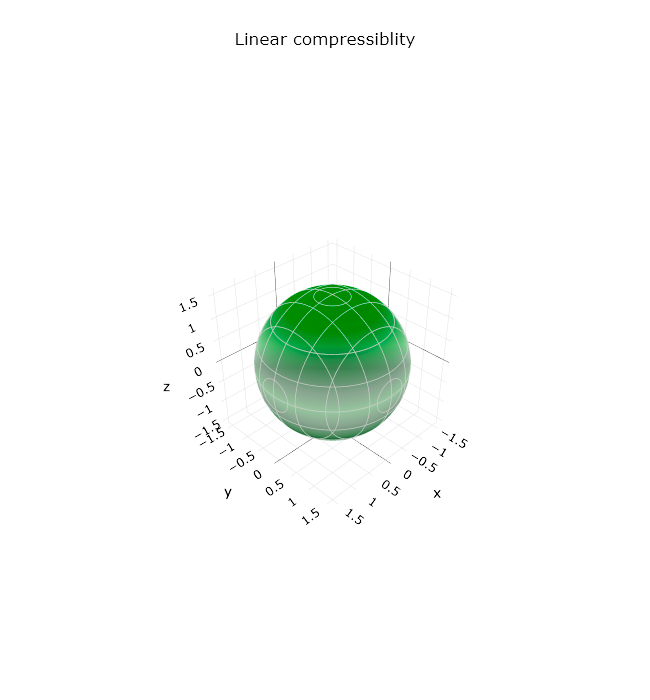 | 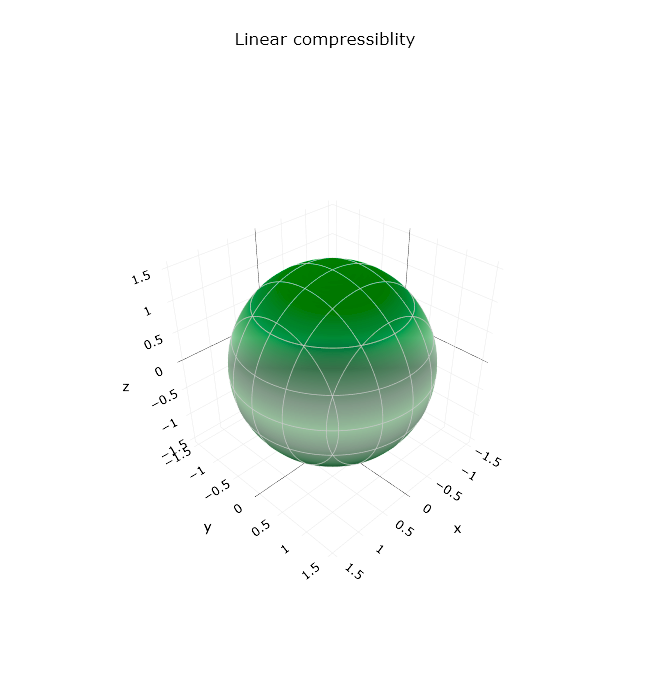 | 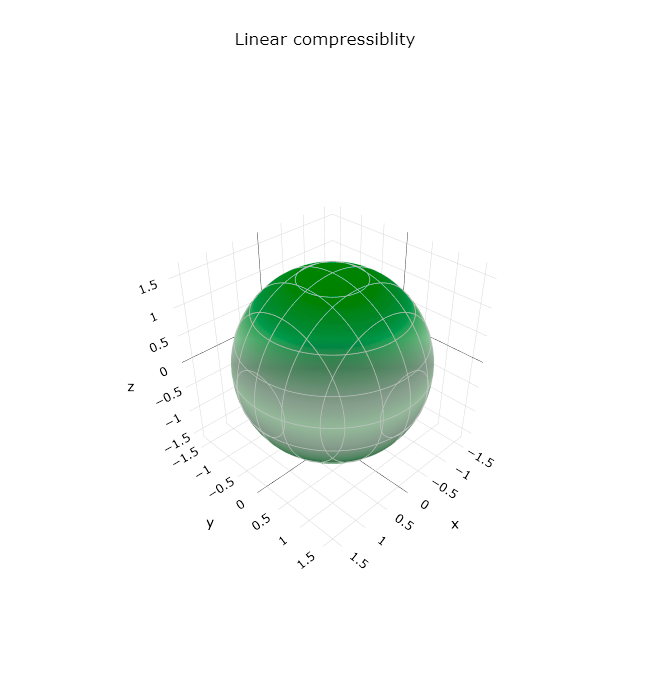 |

**Figure S3:** 3D representation of the directional dependency of *E, G, ν and β* for $NiCo$ generated by (a) Non GB, (b) GB Σ7, and (c) GB Σ9 for ${Ni}_{0.25}{Co}_{0.75}$ alloys.

|  | (a) Non GB | (b) GB Σ7 | (c) GB Σ9 |
| --- | --- | --- | --- |
| Spatial dependence of $E$ | 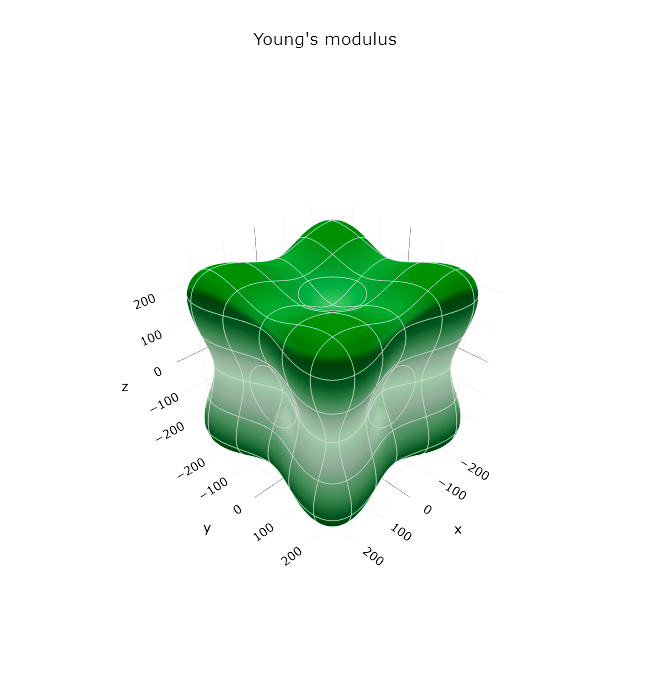 | 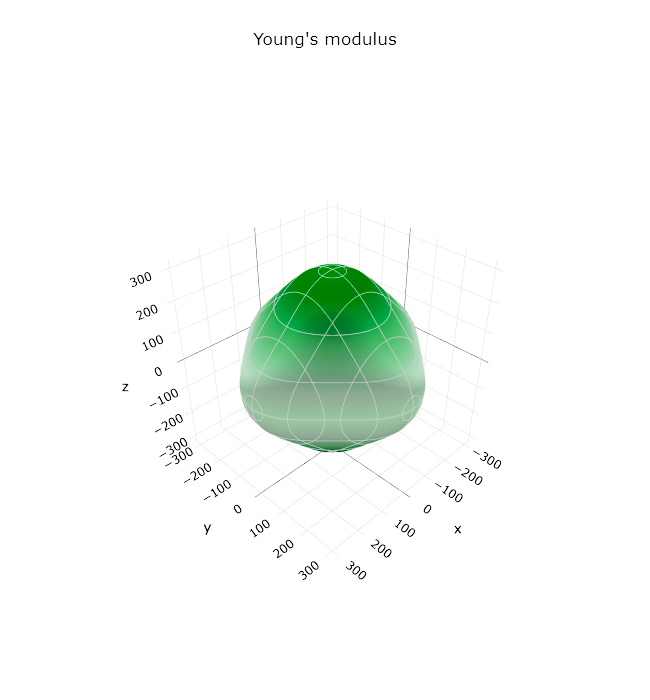 | 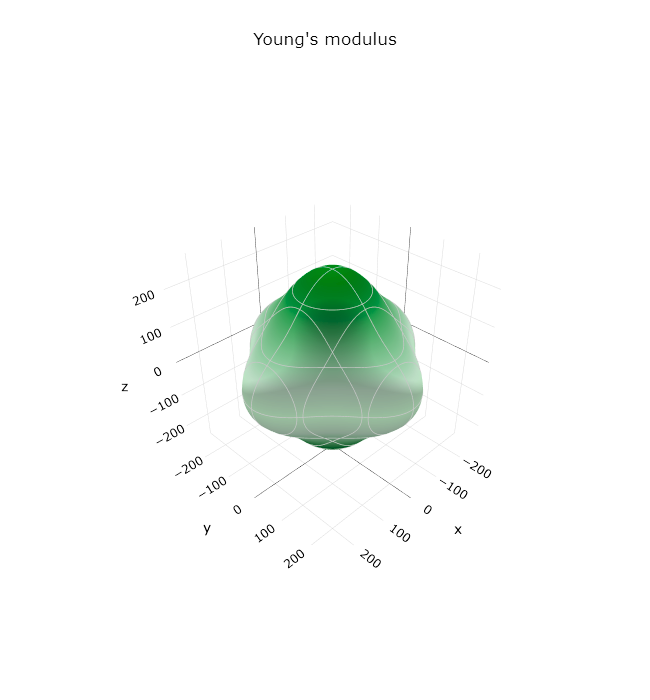 |
| Spatial dependence of $G$ | 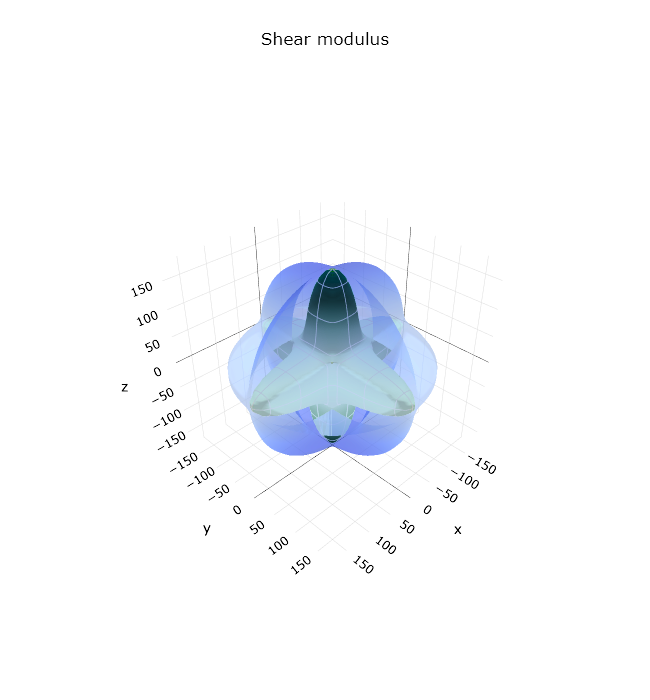 | 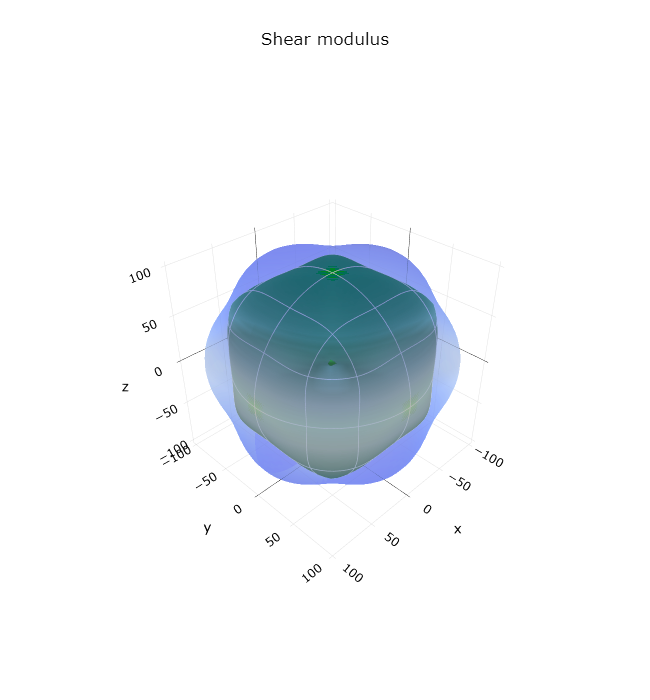 | 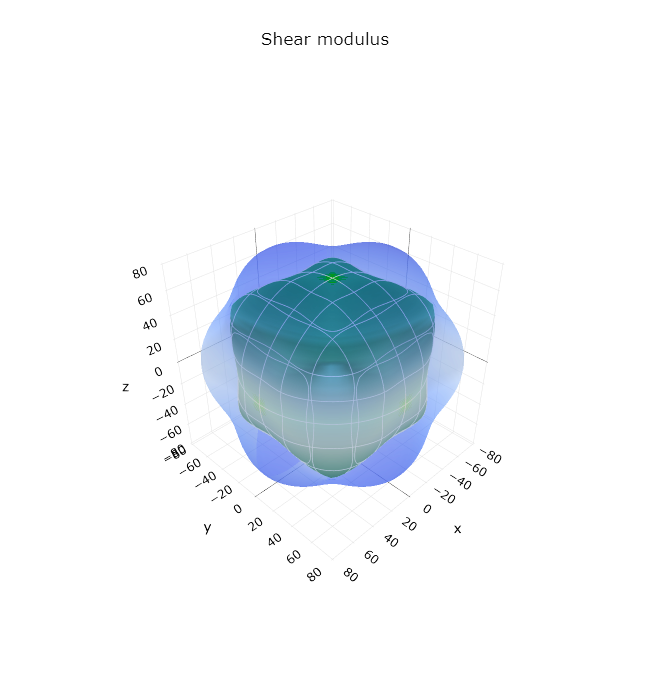 |
| Spatial dependence of $\nu$ | 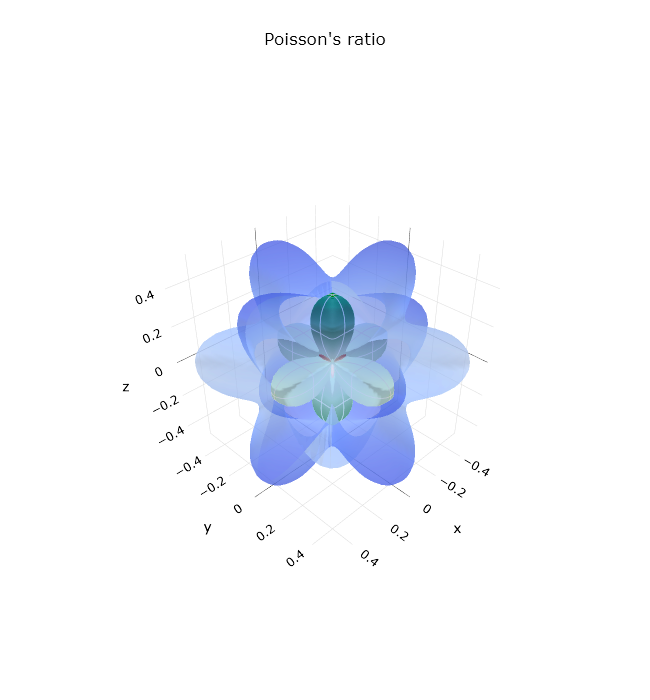 | 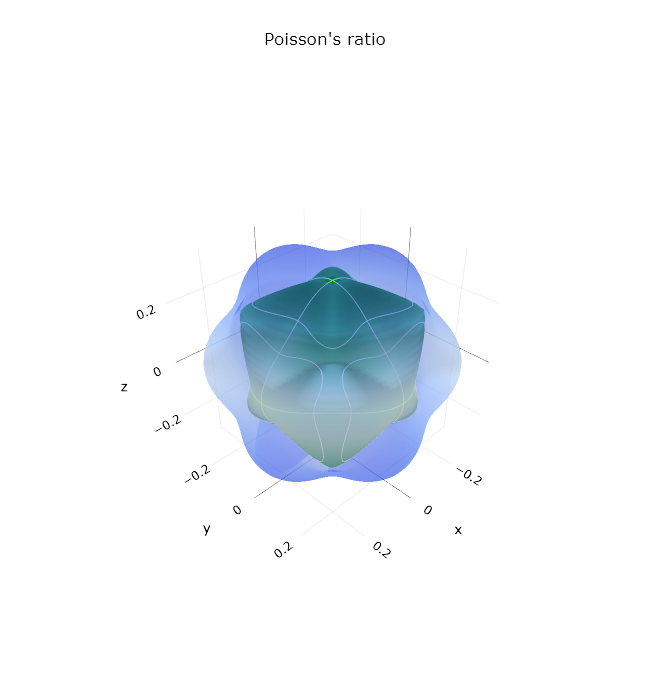 | 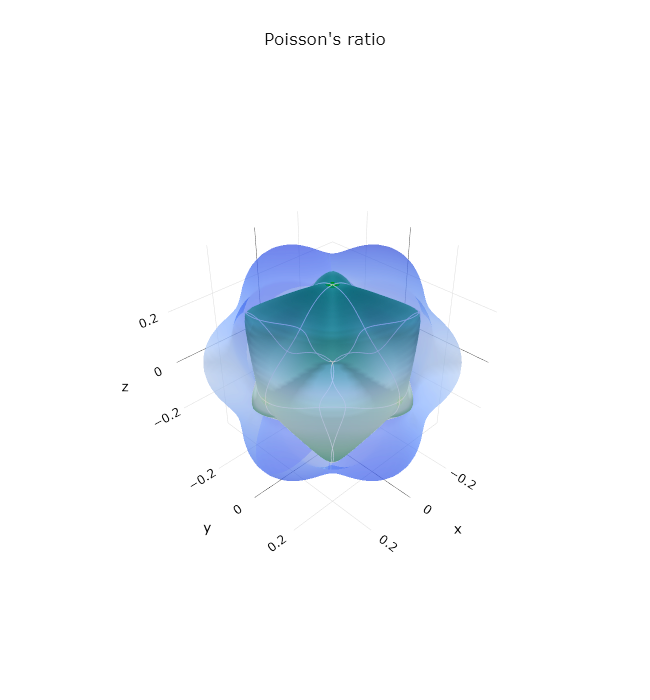 |
| Spatial dependence of $\beta$ | 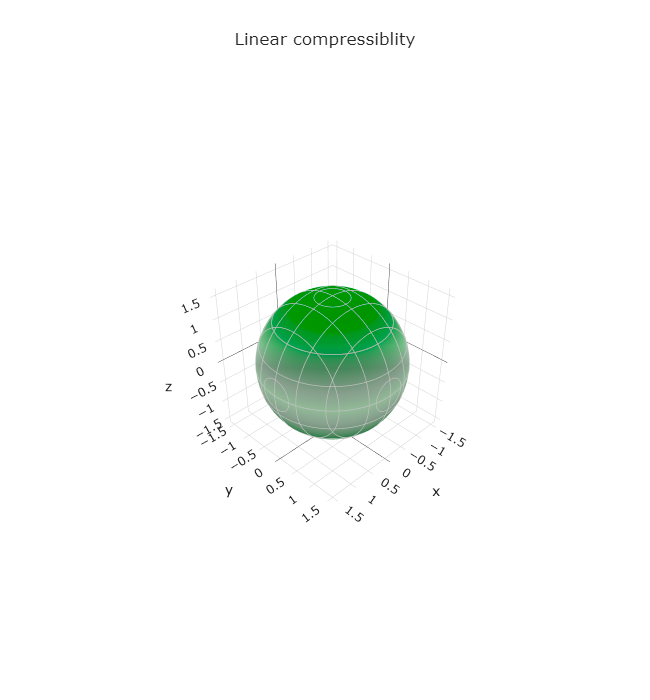 | 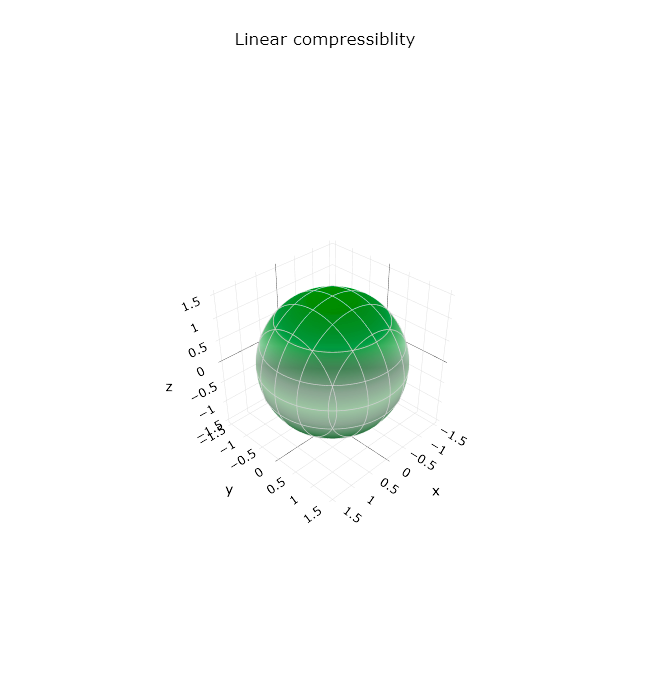 | 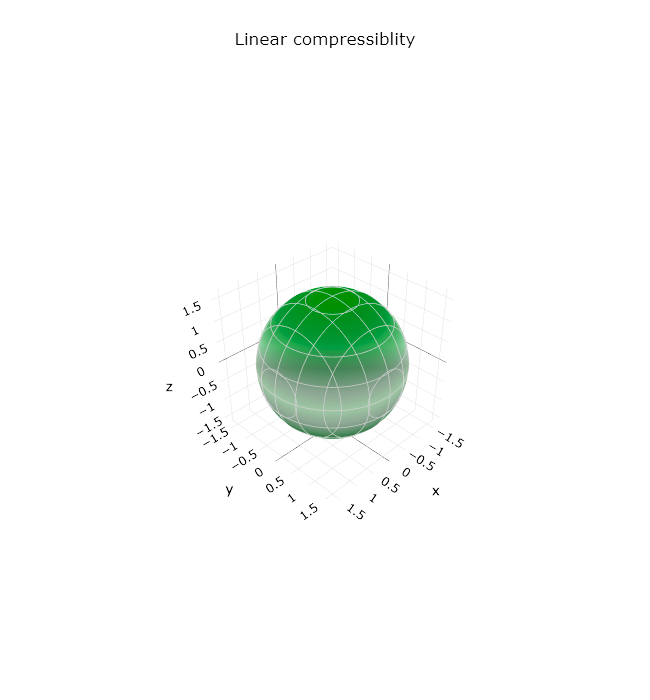 |

**Figure S4:** 3D representation of the directional dependency of *E, G, ν and β* for $NiCo$ generated by (a) Non GB, (b) GB Σ7, and (c) GB Σ9 for ${Ni}_{0.75}{Co}_{0.25}$ alloys.

**References**

1. Tao, Xiaoma, et al. First-principles investigation of the thermo-physical properties of $\mathrm{Ca}_{3}\mathrm{Si}_{4}$. J.Solid State Chem. **194** (2012): 179-187.
2. Hill, Richard. The elastic behaviour of a crystalline aggregate. Proc. Phys. Soci.Sec.A **65** (1952): 349.
3. Li C, Wang Z. First-principles study of structural, electronic, and mechanical properties of the nanolaminate compound $\mathrm{Ti}_{4}\mathrm{GeC}_{3}$ under pressure. J. Appl. Phy.**107 (**2010): 123511.
4. Pettifor, D. G. Theoretical predictions of structure and related properties of intermetallics. Mater. Sci. Tech. **8** (1992): 345-349.
5. Liu SY, Zhang S, Liu S, Li DJ, Li Y, Wang S. Phase stability, mechanical properties and melting points of high-entropy quaternary metal carbides from first-principles. J. Eur. Ceram. Soc. **41 (**2021): 6267-74.
6. Z. Sun, D. Music, R. Ahuja, and J.M. Schneider, Theoretical investigation of the bonding and elastic properties of nanolayered ternary nitrides. Phys. Rev. B , **71 (**2005): 193402.
7. Naher M.I, Naqib SH. Possible applications of Mo2C in the orthorhombic and hexagonal phases explored via ab-initio investigations of elastic, bonding, optoelectronic and thermophysical properties. Res. Phys. **37 (**2022): 105505.
8. Ranganathan, S. I., Ostoja-Starzewski, M. Universal elastic anisotropy index. Phys. Rev. Lett. **101 (**2008): 055504.
9. Frantsevich, I. N., Voronov, F. F., Bakuta, S. A. Handbook on Elastic Constants and Moduli of Elasticity for Metals and Nonmetals. Naukova Dumka: Kiev (1982).
10. Naher, M.I., Naqib, S.H. Possible applications of $\mathrm{Mo}_{2}C$ in the orthorhombic and hexagonal phases explored via ab-initio investigations of elastic, bonding, optoelectronic and thermophysical properties. Res. Phys. **37** (2022): 105505.
11. Kube, C. Elastic anisotropy of crystals, AIP Adv. **6** (2016): 095209.
12. Parvin, F., Naqib, S.H. Pressure dependence of structural, elastic, electronic, thermodynamic, and optical properties of van der Waals-type NaSn2P2 pnictide superconductor: Insights from DFT study. Res. Phys. **21** (2021).
13. Ranganathan, S. I., Ostoja-Starzewski, M. Universal elastic anisotropy index. Phys. Rev. Lett. **101** (2008): 055504.
14. Kube, Christopher M. Elastic anisotropy of crystals. AIP adv. **6** (2016).
